# Data‐Driven Design of Self‐Adhesive Epidermal Electrodes and Sensors

**DOI:** 10.1002/advs.76928

**Published:** 2026-07-31

**Authors:** Xuan Li, Shilei Wang, Milad Razbin, Danish Tahir, Chen Sang, Shuhua Peng, Markus Müllner, Wenlong Cheng, Wei Chen, Chun Hui Wang, Shuying Wu

**Affiliations:** ^1^ School of Aerospace Mechanical and Mechatronic Engineering The University of Sydney Sydney New South Wales Australia; ^2^ School of Biomedical Engineering The University of Sydney Sydney New South Wales Australia; ^3^ School of Mechanical and Manufacturing Engineering The University of New South Wales Sydney New South Wales Australia; ^4^ Key Centre for Polymers & Colloids School of Chemistry The University of Sydney Sydney New South Wales Australia; ^5^ The University of Sydney Nano Institute (Sydney Nano) The University of Sydney Sydney New South Wales Australia

**Keywords:** biopotential, data‐driven, epidermal electrodes, self‐adhesive

## Abstract

Stretchable and self‐adhesive epidermal electrodes and sensors with long‐term stability are highly desirable for wearable applications. These electrodes and sensors typically comprise multiple components, each contributing distinct mechanical and electrical functions. However, their materials design and optimization rely heavily on time‐consuming trial‐and‐error approaches, highlighting the need for a more effective strategy. Here, we report a data‐driven composition optimization strategy integrating artificial neural network (ANN) modeling and genetic algorithm (GA) optimization for the rational design of self‐adhesive epidermal electrodes/sensors. By defining optimization objectives that prioritized either high electrical conductivity and adhesion or high piezoresistive sensitivity, stretchable epidermal electrodes and sensors were developed. The optimized electrode exhibits high stretchability (∼ 177%), robust adhesion (0.10 N cm^−^^1^), and low skin electrode contact impedance (∼ 72 kΩ at 10 Hz), enabling more stable long‐term acquisition of electromyograms (EMG), electrocardiograms (ECG), and electroencephalograms (EEG) signals, compared to commercial Ag/AgCl gel electrodes. The resulting optimal sensor based on a different material composition demonstrates large stretchability (∼ 153%) and good piezoresistive sensitivity (gauge factor ∼ 4.79), enabling motion monitoring and human‐machine interface demonstrations. This work highlights the effectiveness of data‐driven optimization for application‐specific design of wearable devices.

## Graphical abstract

This work presents self‐adhesive, stretchable epidermal electrodes and sensors developed through a data‐driven design framework that integrates artificial neural networks with genetic algorithms. By tailoring optimization objectives, either maximizing electrical conductivity and adhesion or enhancing piezoresistive sensitivity, the study enables the rational design of material compositions, yielding high‐performance skin electrodes and sensors.

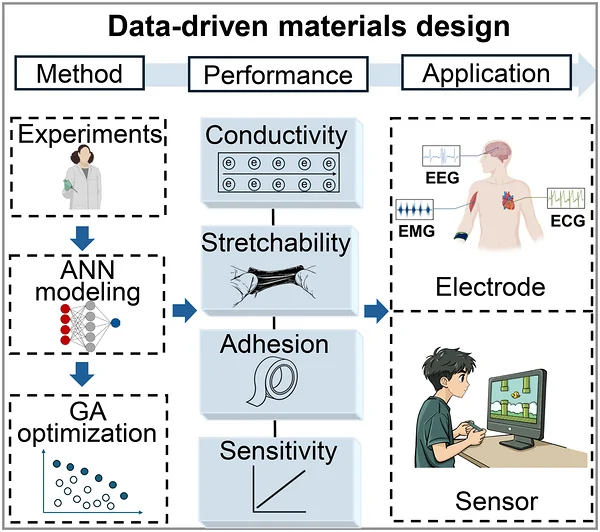

## 1 Introduction

Epidermal electronics enable the noninvasive acquisition of physiological signals. They have therefore been explored for applications in health monitoring, clinical diagnosis, and human‐machine interface systems [1, 2, 3, 4, 5, 6, 7]. A wide range of materials have been investigated for epidermal electronics, including conductive polymers, metallic nanomaterials, elastomers, and hydrogels, which can be processed through printing, casting, coating, or transfer‐based fabrication techniques [8]. Stretchable electrodes and sensors are fundamental components of epidermal electronics. The electrodes must maintain high electrical conductivity under large deformation, exhibit excellent stretchability, and adhere securely to the skin, while the sensors require high sensitivity, fast response, minimal signal drift, and stable performance under repeated strain [9, 10].

Conventional Ag/AgCl gel electrodes are widely used for electrophysiological recording. However, their wet, gel‐based nature leads to skin irritation, dehydration, and degradation of electrode‐skin interfacial stability during prolonged use, limiting their suitability for long‐term monitoring [11, 12]. Recently, conductive hydrogels have been explored as epidermal electrodes for electrophysiological recording, owing to their skin‐like physicochemical properties [13, 14, 15, 16]. Nevertheless, they share a similar limitation as their reliance on water‐containing matrices can result in dehydration‐induced performance deterioration during prolonged use. Dry electrodes based on conductive polymers and nanocomposites have therefore been developed as alternatives. For example, Sinha et al. reported screen‐printed poly (3,4‐ethylenedioxythiophene):poly (styrenesulfonate) (PEDOT: PSS) electrodes on commercial textiles for electrocardiogram (ECG) recording [17]. However, the mechanical compliance was largely constrained by the textile substrate with limited stretchability. Moreover, delamination from the skin during body motion resulted in signal degradation. Zucca et al. developed ultrathin ethyl cellulose/PEDOT: PSS bilayer electrodes that exhibited strong skin adhesion and were successfully used for electromyograms (EMG) monitoring [18]. Yet the restricted stretchability of the bilayer structure made the recorded signals susceptible to motion‐induced artifacts.

Meanwhile, stretchable epidermal sensors have been developed for monitoring human motion and physiological deformation. However, a trade‐off frequently exists among sensitivity, stretchability, and reliable skin adhesion [19]. For example, Lee et al. reported a biodegradable strain sensor exhibiting an ultrahigh gauge factor (∼ 100). But its effective stretchability was limited (∼ 1.0%) [20]. Zheng et al. demonstrated self‐adhesive strain sensors with high stretchability (∼ 600%) and conformal skin adhesion; nonetheless, the gauge factor remained relatively low (< 1.8) [21]. These representative studies suggest that simultaneously realizing high sensitivity, large stretchability, or strong adhesion can be challenging.

Both stretchable epidermal electrodes and sensors are typically constructed from multicomponent composite systems, in which different constituents contribute complementary electrical, mechanical, and interfacial functions. However, when multiple components are involved, the compositional design space becomes large, making conventional trial‐and‐error optimization inefficient and difficult to reproduce. Moreover, the relationships between composition and performance metrics are often highly nonlinear and involve trade‐offs among multiple objectives, further complicating rational design. Data‐driven optimization approaches, such as artificial neural network (ANN) modeling combined with genetic algorithm (GA) optimization, provide an effective strategy to address these challenges [22, 23]. ANN models can capture complex nonlinear relationships between material composition and functional properties, while GA enables efficient exploration of high‐dimensional design spaces to identify optimal solutions under multiple constraints. For example, Liu et al. used an ANN‐GA strategy to design Cu‐based alloy compositions by balancing mechanical strength and electrical conductivity, and experimentally validated the selected formulations [24]. Venkateshwar et al. employed an ANN‐GA strategy to optimize high‐dimensional design variables in graded metal foams for enhanced thermal performance [25]. Despite this, such strategies have rarely been applied to the composition optimization of epidermal electronic materials.

In this study, we report a data‐driven composition design strategy by integrating ANN modeling and GA optimization to engineer multifunctional soft electronic materials. This strategy is applied to the development of self‐adhesive, conductive, and stretchable dry electrodes and sensors based on composites of silver nanowire, PEDOT:PSS, poly(vinyl alcohol), and tannic acid (TA), each of which contributes different functions. Silver nanowires (AgNWs) and PEDOT:PSS provide conductive pathways, PVA serves as a stretchable matrix, while TA is expected to contribute to strong interfacial adhesion with the skin. The material composition was optimized by the ANN‐GA framework. Moreover, their potential as skin electrodes for biopotential measurement (EEG, ECG, EMG) and skin sensors for motion monitoring and human‐machine interface were demonstrated.

## 2 Results and Discussion

### 2.1 Conceptual Design

The materials design, fabrication process, and ANN‐GA guided optimization method are illustrated in Figure 1. The skin electrodes and sensors (denoted by APPT) are made of four components (Figure 1a): AgNWs, PEDOT:PSS, PVA, and TA. Briefly, AgNWs and PEDOT:PSS provide conductive pathways, PVA serves as a stretchable matrix, and TA promotes interfacial adhesion through its abundant phenolic hydroxyl groups, which form reversible hydrogen bonds with polar groups on the skin surface [26, 27]. Different strategies have been employed to enhance the adhesion of the electrodes/sensors to skin, including hydrogen bonding [28], hydrophobic anchoring [29], van der Waals [30], or through creating ultrathin or unique micro/nanostructures (e.g., micro/nanopillar interlocking) that increase the effective contact area [31] (as listed in Tables S1 and S2). The TA‐assisted adhesion was adopted in this work to create self‐adhesive sensors/electrodes, based on a comprehensive consideration of the interactions between the AgNWs/PEDOT:PSS conductive components and PVA, as well as the need to achieve desirable adhesion to skin without the use of additional adhesives.

**FIGURE 1:**
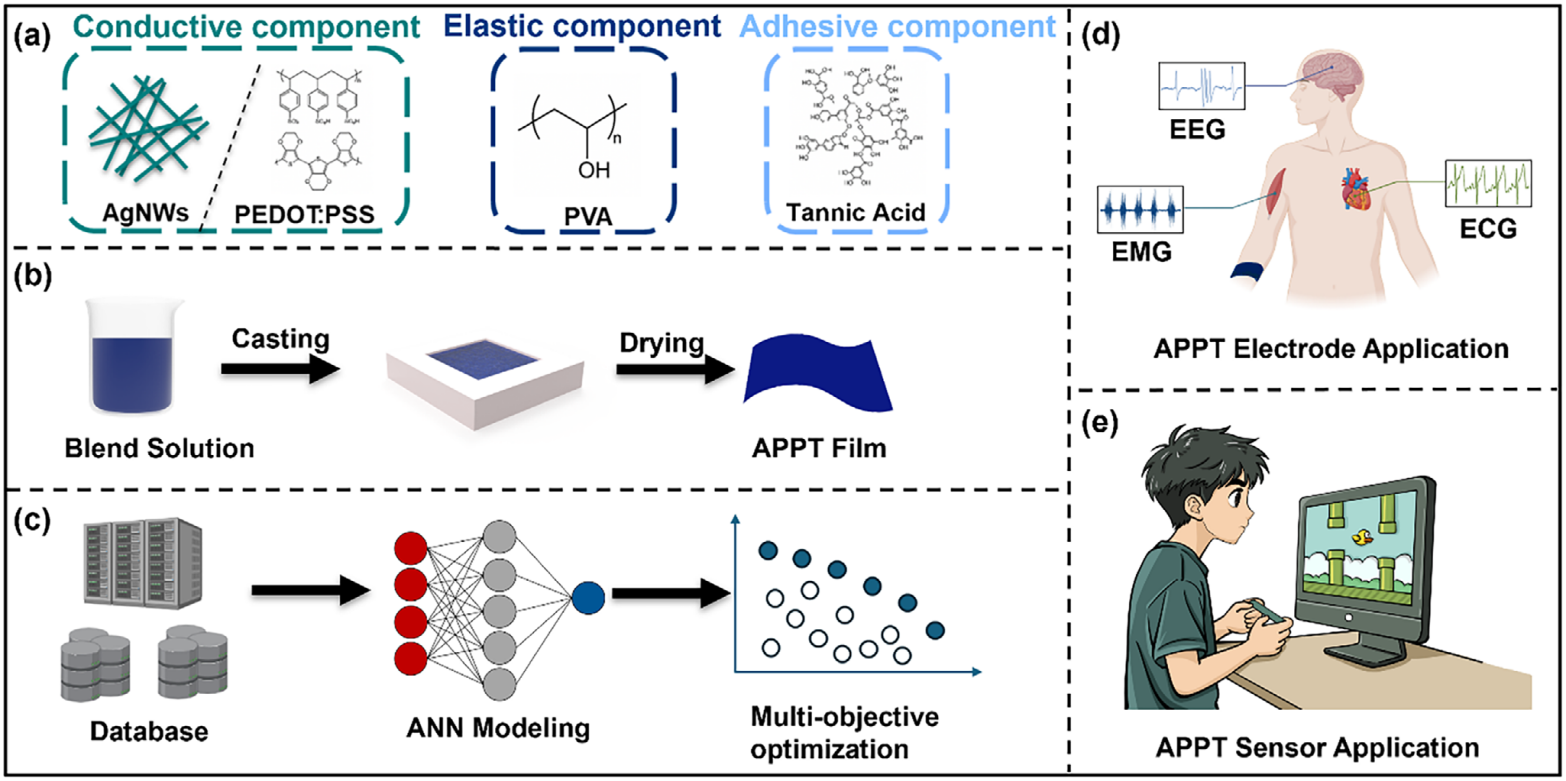
Schematic illustration of the material design, ANN‐GA guided optimization, and bioelectronic applications of the APPT films. (a) Components of APPT and their contribution. (b) Fabrication process of the APPT films. (c) ANN‐GA‐assisted optimization workflow. Schematic illustration of (d) electrophysiological monitoring and (e) human‐interface application.

The APPT films are fabricated through a simple solution casting process (Figure 1b). Figure 1c illustrates the ANN‐GA‐assisted optimization process starting from collecting experimental data to construct a database, which is then used to train an ANN model describing the nonlinear relationship between material composition and target performance metrics. Based on the trained ANN model, a GA is employed to perform multi‐objective optimization and identify optimal material compositions under multiple constraints. The optimized APPT dry electrode is then used for EMG, ECG, and EEG acquisition (Figure 1d). Furthermore, the same strategy is extended to the design of flexible strain sensors, which are then explored for motion monitoring and human‐machine interface applications (Figure 1e).

The performance of the epidermal electrodes/sensors was optimized by systematically controlling the composition of the four components: AgNWs, PEDOT:PSS, PVA, and TA. The weight fraction of these four components was treated as an independent control variable in the design. Four key performance metrics of the resultant composites APPT are considered and identified as important for skin electrode/sensor applications, including electrical conductivity, elongation, adhesion, and gauge factor (piezoresistive sensitivity). The experimental runs were designed using response surface methodology (RSM) to streamline the parametric study and reduce the number of samples required from 286 (based on a conventional trial‐and‐error screening with a 0.1 composition increment under the mixture constraint ∑x_i_ = 1) to 24 (as outlined in Table S3). The compositional ranges of each component were determined based on preliminary trial‐and‐error experiments. A quadratic model based on a single‐block I‐optimal mixture design was employed, with the experimental runs generated through a randomized procedure and without the need for additional data points. The selected formulations and their corresponding performance metrics are summarized in Table S4. Overall, the 24 designed experiments effectively represented the original 286 candidate formulations, significantly reducing experimental time and material consumption.

### 2.2 Modeling

To establish quantitative relationships between material composition and composite performance, ANNs were employed owing to their strong capability in modeling complex nonlinear multivariable systems. The weight fractions of AgNWs, PEDOT: PSS, PVA, and TA were used as input variables, while conductivity, elongation, adhesion, and gauge factor were treated as output responses. A feed‐forward ANN with a single hidden layer was constructed and trained using the Levenberg‐Marquardt algorithm [32]. Prior to model training, all experimental data were normalized to ensure numerical stability and efficient convergence. The dataset comprising 24 experimental runs was randomly divided into training and testing sets with a ratio of 9:3. To assess model robustness, six‐fold cross‐validation was performed within the training dataset, and the detailed fold‐wise results are provided in Tables S5–S8. The mean validation coefficient of determination (R^2^) values obtained from the six folds were 0.980 ± 0.017 for conductivity, 0.953 ± 0.032 for elongation at break, 0.985 ± 0.016 for adhesion, and 0.957 ± 0.012 for gauge factor. The corresponding mean squared error (MSE) values were 0.00387 ± 0.00329, 0.00997 ± 0.00689, 0.00819 ± 0.00861, and 0.00861 ± 0.00233, respectively, based on normalized response values. All validation folds showed R^2^ values higher than 0.925, indicating that the models retained good predictive performance for samples excluded from the corresponding training folds.

To evaluate the predictive performance of the ANN model, the total goodness function (TGF) was adopted as a comprehensive metric, integrating the effects of data split ratio, R^2^, and MSE, as reported in previous studies [33, 34, 35, 36]. By simultaneously accounting for fitting accuracy and prediction robustness, the TGF provides an effective criterion for assessing model reliability. Based on systematic hyperparameter optimization, an optimal network architecture with six neurons in the hidden layer was identified. The detailed modeling procedures, mathematical formulations, normalization schemes, and hyperparameter settings are provided in Section S1.

The predictive capability of the ANN model was evaluated by comparing the predicted and experimental values for both the training and testing datasets, as illustrated in Figure 2a–h. The parity plots show that the predicted results closely follow the experimental measurements for all four properties, with correlation coefficients (R^2^) consistently above 0.90 during both training and testing. These results indicate that the ANN model can effectively capture the complex, multiparametric interactions among the components and provides improved generalization performance across both seen and unseen formulations.

**FIGURE 2:**
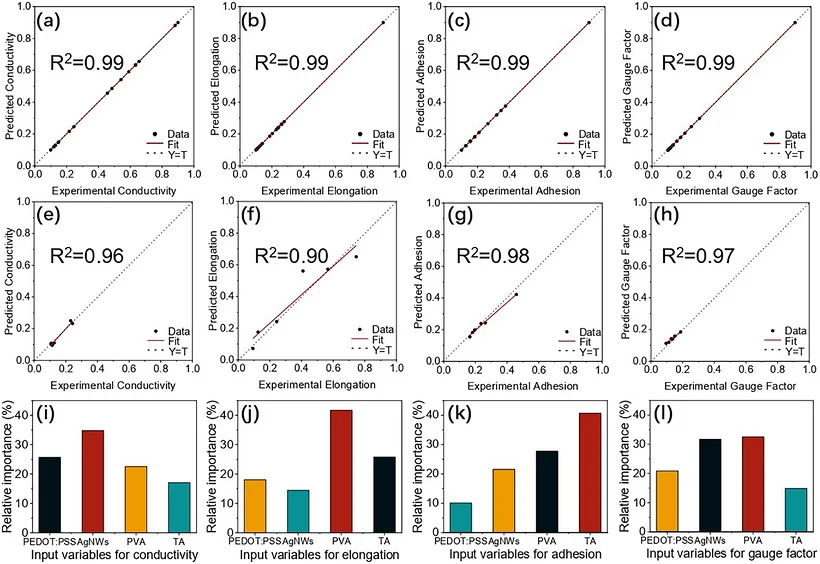
Comparison between experimental and model‐predicted values for the APPT films and relative significance of input variables. Parity plots of experimental vs. predicted values for the four target properties during the training stage: (a) conductivity, (b) elongation at break, (c) adhesion (maximum peel strength), and (d) gauge factor; during the testing stage: (e) conductivity, (f) elongation at break, (g) adhesion, and (h) gauge factor. Feature‐importance (sensitivity) analysis showing the relative significance of each input variable for (i) conductivity, (j) elongation at break, (k) adhesion, and (l) gauge factor.

In addition to prediction accuracy, the relative significance of each input component on the four target outputs was analyzed, as shown in Figure 2i–l. The sensitivity analysis reveals distinct influence patterns for each property. For example, AgNWs exhibit the strongest contribution to conductivity, whereas PVA and TA play more dominant roles in determining elongation at break and adhesion strength. The gauge factor is primarily influenced by the content of AgNWs and PVA, highlighting the interplay between conductive network density and polymer matrix composition. This variable‐importance analysis further elucidates the structure‐property relationships within the APPT composite and provides valuable insights for formulation optimization.

### 2.3 Optimization Process

To identify the optimal formulation of the APPT for skin electrode and/or sensor application, a GA was employed in conjunction with the trained ANN models.

For the electrode application, electrical conductivity and interfacial adhesion were defined as the primary optimization objectives, while elongation at break and gauge factor were imposed as performance constraints to ensure mechanical compliance and signal stability. The ANN‐predicted properties were used to construct a constrained fitness function, and the GA was applied to efficiently explore the compositional design space. The optimization yielded a set of high‐performance formulations that balance conductivity and adhesion under the prescribed constraints, from which an optimal composition was selected for experimental validation. Detailed descriptions of the fitness function formulation and GA implementation are provided in Section S2.

Figure 3a shows the Pareto front obtained from the GA identified candidate formulations, illustrating the trade‐off between electrical conductivity and interfacial adhesion under the imposed constraints. The non‐dominated solutions collectively define the achievable performance envelope of the APPT electrodes. Among these solutions, the knee point (highlighted in red in Figure 3a) was selected as the optimal formulation, as it represents a balanced compromise between conductivity and adhesion, where further improvement in one objective would lead to a disproportionate deterioration in the other. The knee‐point criterion is widely adopted in multi‐objective optimization as a rational decision strategy for balancing competing objectives [37, 38, 39]. In the present study, this formulation simultaneously provides high electrical conductivity for low impedance signal acquisition and strong interfacial adhesion for stable skin contact, both of which are essential requirements for epidermal electrodes. The optimal composition determined from the knee point consists of 15% PEDOT:PSS, 8% AgNWs, 10% PVA, and 67% TA (Table 1).

**FIGURE 3:**
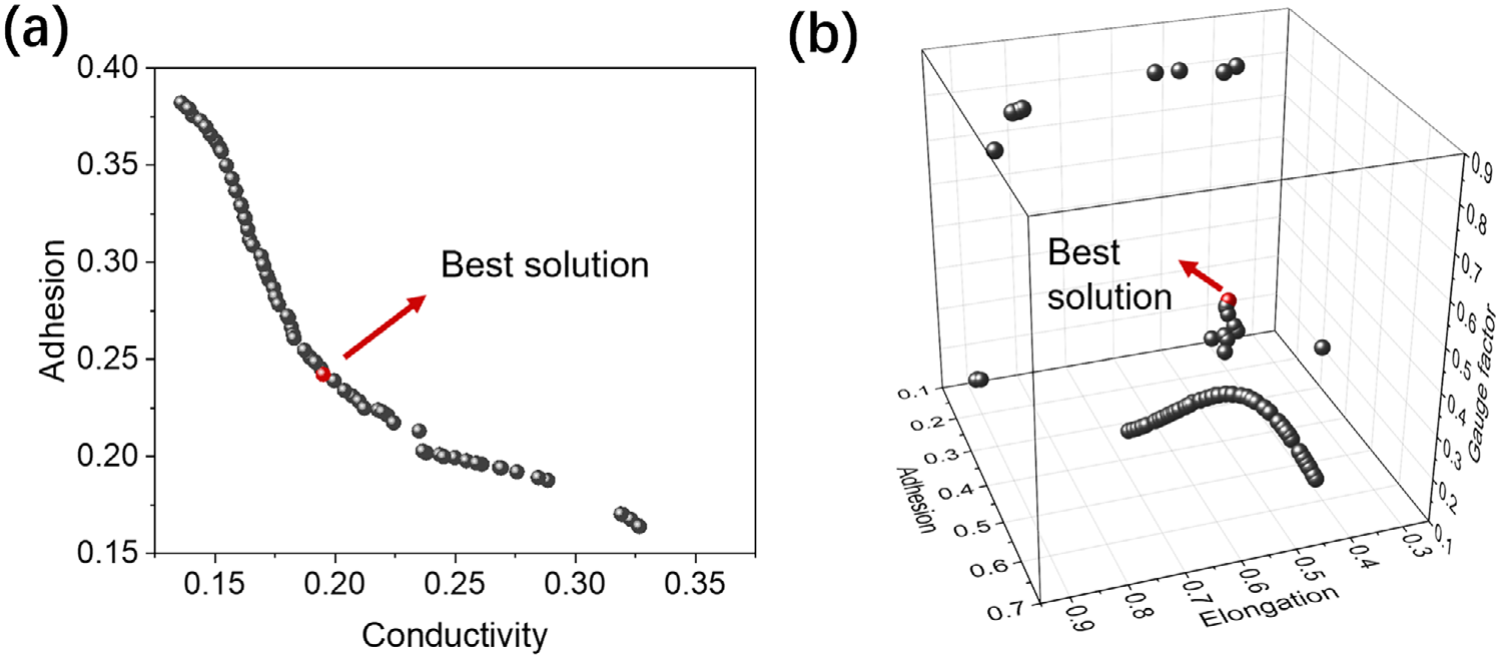
Multi‐objective optimization results. (a) Pareto front obtained by balancing adhesion and conductivity for APPT electrodes. (b) Three‐dimensional Pareto front obtained by optimizing elongation, adhesion, and gauge factor for APPT sensors.

**TABLE 1:** Optimized compositions for epidermal electrodes and sensors.

Optimized compositions	Epidermal electrodes	Epidermal sensors
PEDOT:PSS	15 wt.%	6 wt.%
AgNWs	8 wt.%	15 wt.%
PVA	10 wt.%	14 wt.%
TA	67 wt.%	65 wt.%

Similarly, the methodology was used for optimizing composite composition for sensors with an optimal combination of gauge factor (sensitivity), stretchability, and interfacial adhesion. As shown in Figure 3b, the Pareto front represents the set of non‐dominated solutions generated by the ANN‐GA framework, reflecting the inherent trade‐offs among elongation, adhesion, and gauge factor. The best compromise solution was selected by simultaneously considering these three objectives to achieve balanced mechanical robustness, strong interfacial adhesion, and high sensing sensitivity, which is suitable for practical wearable sensor applications [40, 41]. The optimal composition consists of 15 wt.% AgNWs, 6 wt.% PEDOT: PSS, 14 wt.% PVA, and 65 wt.% TA (Table 1).

### 2.4 Characterization of APPT Electrodes

The electrical performances (electrical conductivity and its variation under tensile strain) and mechanical properties (tensile and adhesive properties) of the optimized APPT electrode were systematically evaluated. The APPT electrode exhibits an electrical conductivity of 16.5 S cm^−^^1^, measured by the 4‐point probe method. Moreover, the impedance of the electrode was examined by the dual‐electrode method along the through‐the‐thickness direction using an LCR meter. As shown in Figure S2, the APPT electrode exhibits substantially lower normalized impedance (∼ 8 Ω) across the measured frequency range (1–10^4^ Hz) compared with a commercial Ag/AgCl gel electrode (> 300 Ω). Furthermore, the skin–electrode contact impedance was measured using two APPT electrodes attached to a volunteer's forearm with a spacing of 1 cm. As shown in Figure 4a,b, the APPT electrodes exhibit lower contact impedance than the Ag/AgCl gel electrodes under dry skin conditions. At 10 Hz, the contact impedance of the APPT electrodes is approximately 72 kΩ, compared with 76 kΩ for the Ag/AgCl gel electrodes. To simulate perspiration conditions, a 0.15 M mNaCl aqueous solution was uniformly sprayed to the skin surface prior to the measurement. The APPT electrodes remained firmly attached to wet skin, and the contact impedance further decreased to approximately 65 kΩ at 10 Hz, slightly lower than that measured under dry conditions. This is because water can wet the skin surface and thus improve the contact between the APPT electrodes and the rough skin surface [42].

**FIGURE 4:**
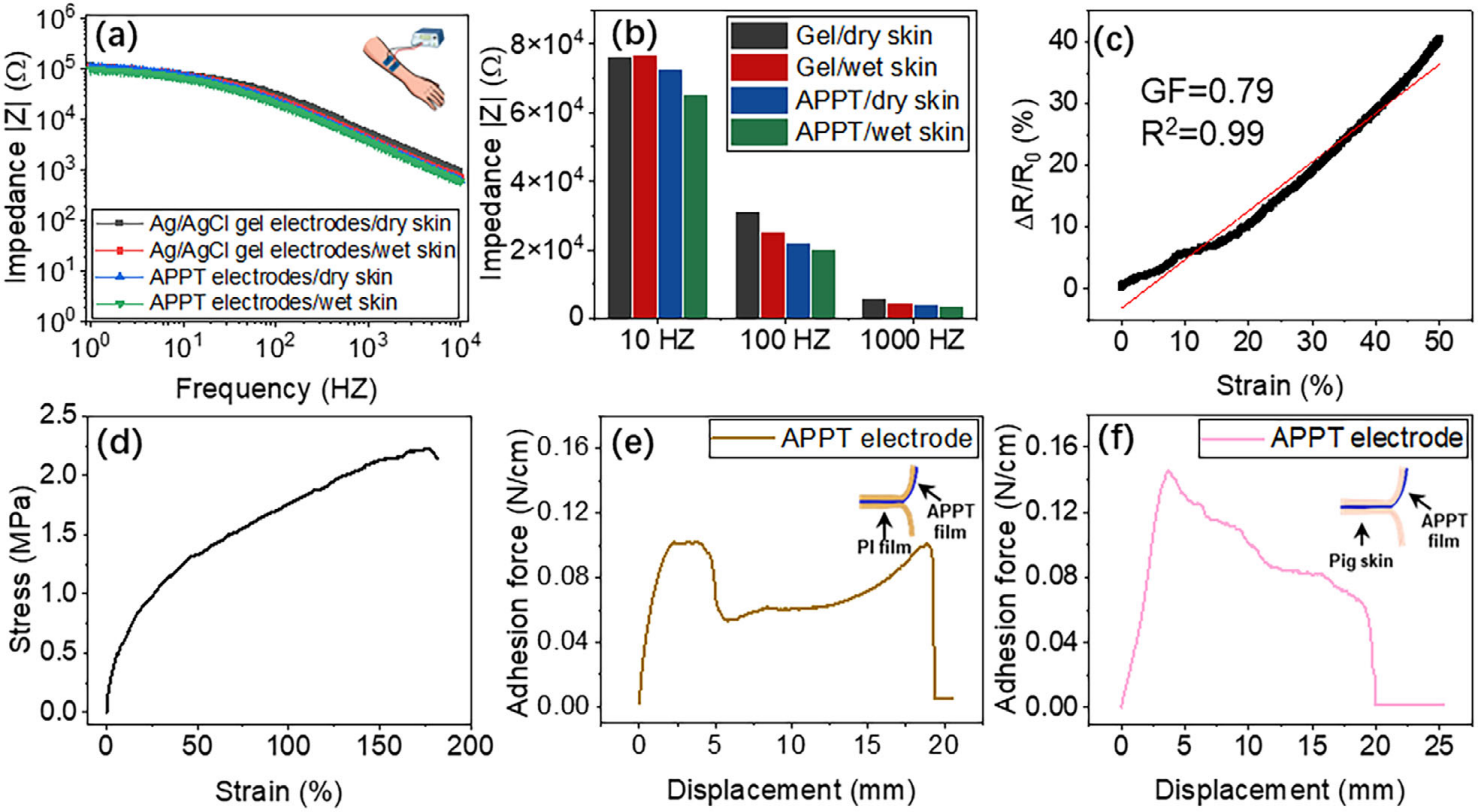
Characterization of APPT electrodes. (a) Impedance spectra of APPT and Ag/AgCl gel electrodes measured on dry and wet skin. (b) Comparison of impedance values at 10, 100, and 1000 Hz under different electrode–skin conditions. (c) Variations of the resistance of the APPT electrode with 50% strain. (d) Stress–strain curve of the APPT electrode. 180° peeling test results comparing the interfacial adhesion force of the APPT electrode on (e) PI film and (f) pig skin.

The relative resistance changes of the APPT electrode under tensile train up to its failure strain are presented in Figure S3, while Figure 4c shows the data up to 50% strain. At low strain, ΔR/R_0_ increases gradually, while a faster increase is observed at higher strain, reaching over 400% at ∼180% strain. Moreover, a nearly linear relative resistance change‐strain response was observed up to ∼50% strain. The slope obtained by linear regression indicates a gauge factor (GF = ΔR/R_0_/strain) of approximately 0.79. This low gauge factor indicates stable electrical conductivity under mechanical deformation, which is desirable for epidermal electrode applications. The durability of the electrode under cyclic deformation was also evaluated by subjecting it to repeated stretching‐releasing tests at 50% strain. As shown in Figure S4, the APPT electrode maintains stable resistance responses over multiple cycles (tested up to 1000 cycles), demonstrating good electromechanical stability and structural integrity under repeated mechanical loading.

Furthermore, the tensile properties of the APPT electrode were investigated. As shown in Figure 4d, it exhibits a tensile strength of 2.22 MPa and a high elongation at break of approximately 177%, indicating excellent stretchability. This stretchability significantly exceeds the typical strain range of human skin (∼ 30%), ensuring reliable mechanical compliance for epidermal applications [43]. Interfacial adhesion strength was another critical property as it governs the quality of skin‐electrode contact and is essential for stable biopotential signal acquisition. In this work, 180° peeling tests were conducted on different substrates. Figure 4e shows a representative peeling force‐displacement curve of the APPT electrode attached to PI film. The electrode exhibits a maximum peeling strength of 0.10 N cm^−^^1^. The adhesion performance was further evaluated on pig skin (Figure 4f). Similarly, the APPT electrode maintains a sustained and stable peeling behavior, with a maximum peeling force of 0.14 N cm^−^^1^, demonstrating its ability to conform to soft and compliant biological substrates. The robust self‐adhesive property of the APPT electrode was further verified by qualitative adhesion demonstrations on various substrates (Figure S5). The APPT electrode can adhere to different substrates, including a glass slide, a Petri dish, and a rubber mold, while supporting weights up to 13.3 g. These results demonstrate the self‐adhesive capability of the electrode across substrates. The good adhesion across substrates with markedly different mechanical properties is attributed to the combined contributions of PVA and TA, where TA provides strong interfacial interactions with skin through hydrogen bonding, while the soft PVA matrix facilitates energy dissipation during peeling [26, 44, 45].

Finally, the experimentally measured properties, including conductivity, elongation at break, adhesion, and gauge factor, were compared with the ANN‐GA predicted results (Figure S6). The deviations between experimental and predicted values are within 14%, confirming the reliability of the ANN‐GA modeling and optimization strategy [46]. Moreover, compared with some recently reported self‐adhesive epidermal electrodes (Table S1), the APPT electrode provides a competitive balance of conductivity, stretchability, and peel adhesion, enabling low skin–electrode impedance and reliable bipotential acquisition. Its dry film design also avoids dehydration and the self‐adhesion avoids the use of a separate adhesive layer.

### 2.5 Application Demonstration of APPT Electrodes for Electrophysiological Signal Detection

This section demonstrates the promising performance of APPT as a skin electrode in biopotential monitoring, enabled by its low interfacial impedance and excellent self‐adhesive properties. For EMG signal acquisition, two square APPT electrodes (2 cm × 1 cm) were attached to a volunteer's forearm with an inter‐electrode spacing of 1 cm (Figure 5a and Video S1). Commercial Ag/AgCl gel electrodes with identical dimensions were used as references. The gripping force was controlled using a digital hand dynamometer. As shown in Figure 5b, the EMG signal amplitude increases progressively with increasing gripping force. The EMG signal intensity was quantified using the root‐mean‐square value. The results in Figure 5b,c reveal a clear correlation between muscle contraction strength and gripping force, with stronger contractions generating higher EMG signal intensities. In addition, the APPT electrodes can resolve low‐amplitude EMG signals induced by flexion and extension of individual fingers (Figure 5d,e), highlighting their high sensitivity and spatial resolution.

**FIGURE 5:**
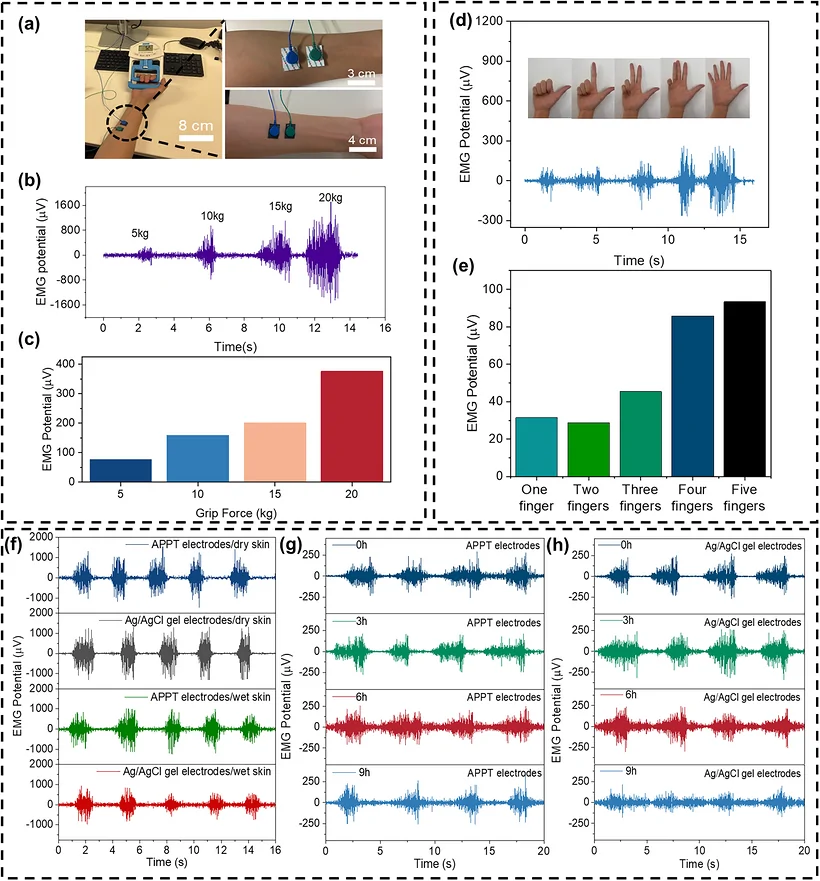
EMG measurements. (a) Photograph showing the attachment of APPT electrodes and commercial Ag/AgCl gel electrodes on the forearm for EMG signal acquisition. (b) EMG signals collected under different gripping forces. (c) Quantified EMG signal intensities extracted under different gripping forces. (d) EMG signals elicited by flexion and extension of different fingers and (e) the corresponding EMG signal intensities. (f) The obtained representative EMG signals recorded on dry and/or wet skin under a gripping force of 10 kg. Long‐term stability test using (g) APPT electrodes and (h) Ag/AgCl gel electrodes under a gripping force of 5 kg up to 9 h of wear (Participant 1).

Furthermore, EMG signals were recorded under five repeated gripping cycles with a gripping force of 10 kg (Figure 5f). The APPT electrodes exhibit signal quality comparable to that of the commercial Ag/AgCl gel electrodes, demonstrating reliable EMG acquisition capability. Notably, owing to their strong self‐adhesiveness and conformal skin contact, the APPT electrodes maintain high‐quality EMG recordings under both dry and wet skin conditions, whereas the Ag/AgCl gel electrodes exhibit pronounced signal degradation on wet skin. This degradation may be attributed to instability at the gel‐skin interface, including gel dilution and impedance fluctuations under moist conditions [47, 48].

The long‐term stability of EMG recordings was further evaluated in three participants and compared with commercial Ag/AgCl gel electrodes. During a standardized gripping task at 5 kg, EMG signals were recorded after 0, 3, 6, and 9 h of continuous electrode wear. Representative results are shown in Figure 5g,h, while the results obtained from the other two participants are provided in Figures S7 and S8. Across all participants, the APPT electrodes maintained distinguishable EMG bursts throughout the 9 h wear period. In contrast, the Ag/AgCl gel electrodes exhibited increased baseline fluctuations and reduced signal clarity after prolonged wear, with the most evident degradation observed at 9 h. These results suggest that the self‐adhesive APPT electrodes provide more stable electrode–skin coupling during prolonged wear than commercial Ag/AgCl gel electrodes. 180° peeling tests were performed to evaluate interfacial adhesion at 0 h and after 9 h of skin attachment (Figure S9). The Ag/AgCl gel electrodes exhibit a substantial reduction in adhesion after prolonged wear (max adhesion force reduced by 81%), whereas the APPT electrodes retain more stable adhesion (max adhesion force reduced by 27%), supporting their improved long‐term recording performance. It should be noted that the decrease in adhesion for APPT may be caused by changes at the electrode–skin interface during prolonged wear, including sweat, skin secretions, dust, skin debris accumulation, and repeated micro‐movements, all of which can reduce the effective contact area.

The performance of the APPT electrodes in measuring ECG, EOG, and EEG was also examined. To record ECG signals, three APPT electrodes of square shape and the same size were used, with two being symmetrically positioned on the inner wrists of the left and right arms and another one being attached to the back of the left hand as the ground electrode (Figure 6a). As shown in Figure 6b,c, the APPT electrodes yield high‐quality ECG signals with well‐resolved P, Q, R, S, and T waveforms and a peak‐to‐peak QRS complex amplitude of approximately 0.19 mV. These ECG waveforms are highly consistent with those obtained using standard Ag/AgCl gel electrodes (Figure 6b,c). Furthermore, EEG signals were recorded using APPT electrodes and compared with those recorded using commercial Ag/AgCl gel electrodes. One electrode was placed on the right side of the forehead, while the reference electrode was positioned behind the ear (Figure 6d). The EEG signals recorded using different electrodes are shown in Figure 6e,f. As shown in Figure 6g, a strong linear correlation was obtained, with a Pearson correlation coefficient of 0.995, verifying that APPT electrodes enable reliable and accurate acquisition of EEG signals comparable to conventional electrodes. For EOG measurement, one electrode was placed on the right of the right eye while the other one on the left eye, with a reference electrode positioned behind the ear (Figure 6h). Eye blinking‐induced EOG signals were clearly captured, and reproducible signal patterns were observed during repeated blinking events, demonstrating stable electrode skin coupling and high sensitivity to ocular potential variations (Figure 6i and Video S2).

**FIGURE 6:**
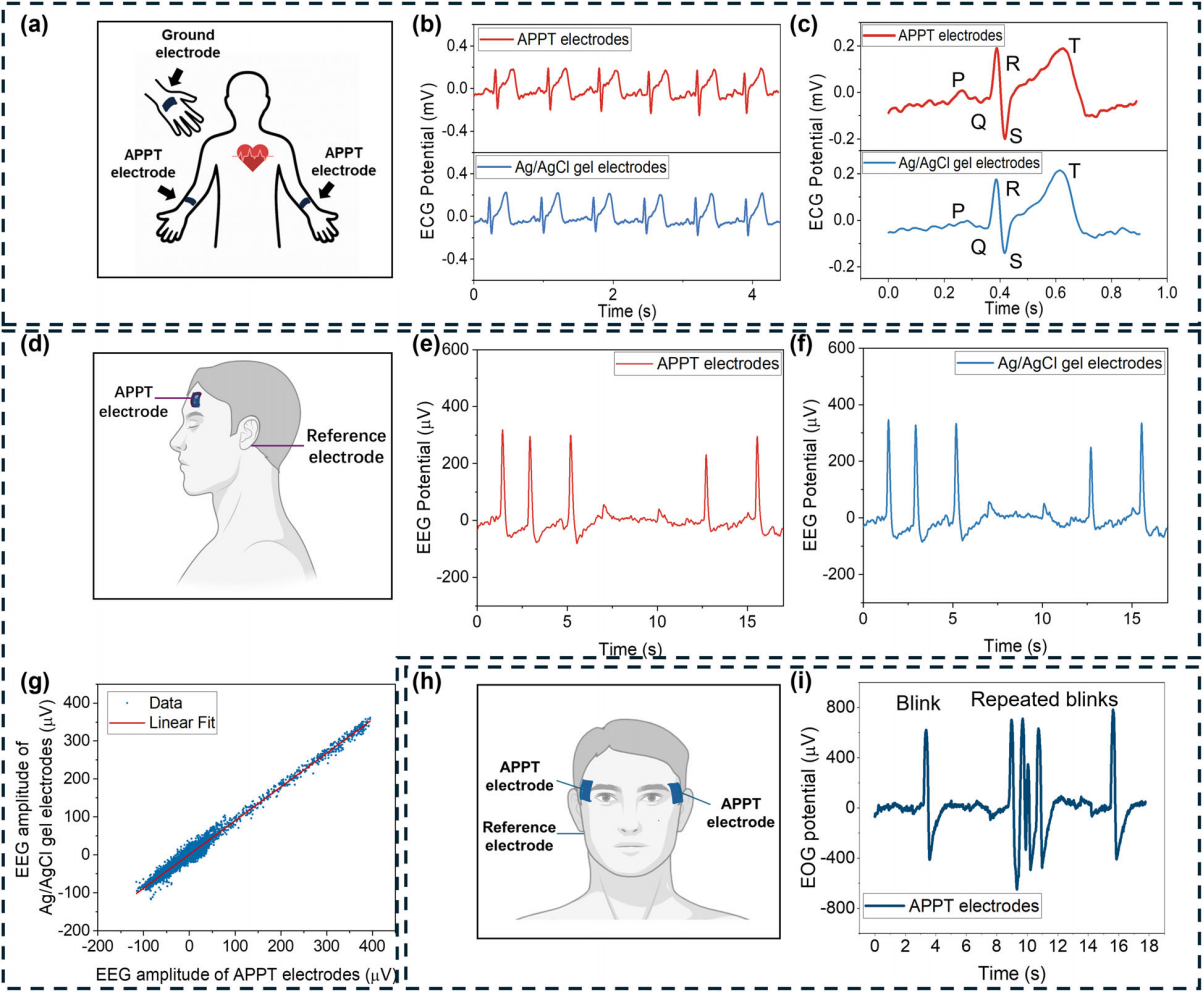
ECG, EEG, and EOG measurements. (a) Schematic illustration of the electrode configuration and measurement setup for ECG recording. (b) Representative ECG signals recorded using APPT electrodes and Ag/AgCl electrodes. (c) Enlarged view of the ECG waveform highlighting the characteristic P, Q, R, S, and T waves. (d) Schematic illustration of the electrode configuration for EEG recording. Representative EEG signals recorded using (e) APPT electrodes and (f) Ag/AgCl electrodes. (g) Correlation analysis between EEG signals simultaneously recorded using APPT electrodes and commercial Ag/AgCl gel electrodes. (h) Schematic illustration of the electrode configuration for EOG recording. (i) Representative EOG signals recorded using APPT electrodes.

### 2.6 Characterization of APPT Sensors

The optimized APPT sensor was created and thoroughly tested. Based on the tensile stress–strain curve (Figure S11), the sensor exhibited a high elongation at break of 153% and a tensile strength of 3.76 MPa. The relative changes in the resistance‐strain curve up to its failure strain were shown in Figure S12. Good linearity was observed in the low strain range up to 50% strain, and a slope (gauge factor) of 4.87 with R^2^ = 0.97 was obtained by linear fitting (Figure 7a). Figure 7b shows the relative resistance change (ΔR/R_0_) of the APPT sensor under cyclic stretching–releasing tests with peak strains of 5%, 10%, 15%, and 20%, all within the elastic deformation regime. The resistance increased monotonically with increasing strain and returned to its initial value upon releasing the strain, indicating stable and reversible piezoresistive behavior. Long‐term cyclic stability was further assessed by subjecting the sensor to 1000 repeated stretching‐releasing cycles at 15% strain, during which negligible variation in sensitivity was observed (Figure 7c). Moreover, its response and recovery times were measured. As shown in Figure 7d, the response time and recovery time were approximately 340 and 170 ms, respectively, comparable to many of the reported sensors [49, 50]. The piezoresistive mechanism of the APPT sensor is illustrated in Figure 7e and believed to be similar to that reported previously [51]. The SEM image in Figure S10a reveals an AgNW‐rich conductive layer with AgNWs distributed across the sensor surface. The higher magnification SEM (Figure S10b) indicates AgNWs are randomly oriented with numerous overlaps and intersections, forming an interconnected conductive network. Previous studies of AgNWs/PEDOT:PSS hybrid conductors have shown that PEDOT:PSS can bridge neighboring AgNWs [52, 53]. Upon stretching, the PVA/TA matrix transfers the applied strain, resulting in increased inter‐nanowire spacing and reduced overlapping area/contacts. The conductive junctions are therefore disrupted, increasing the junction resistance. Upon releasing the load, the restoration of the conductive junctions contributes to the recovery of the conductivity.

**FIGURE 7:**
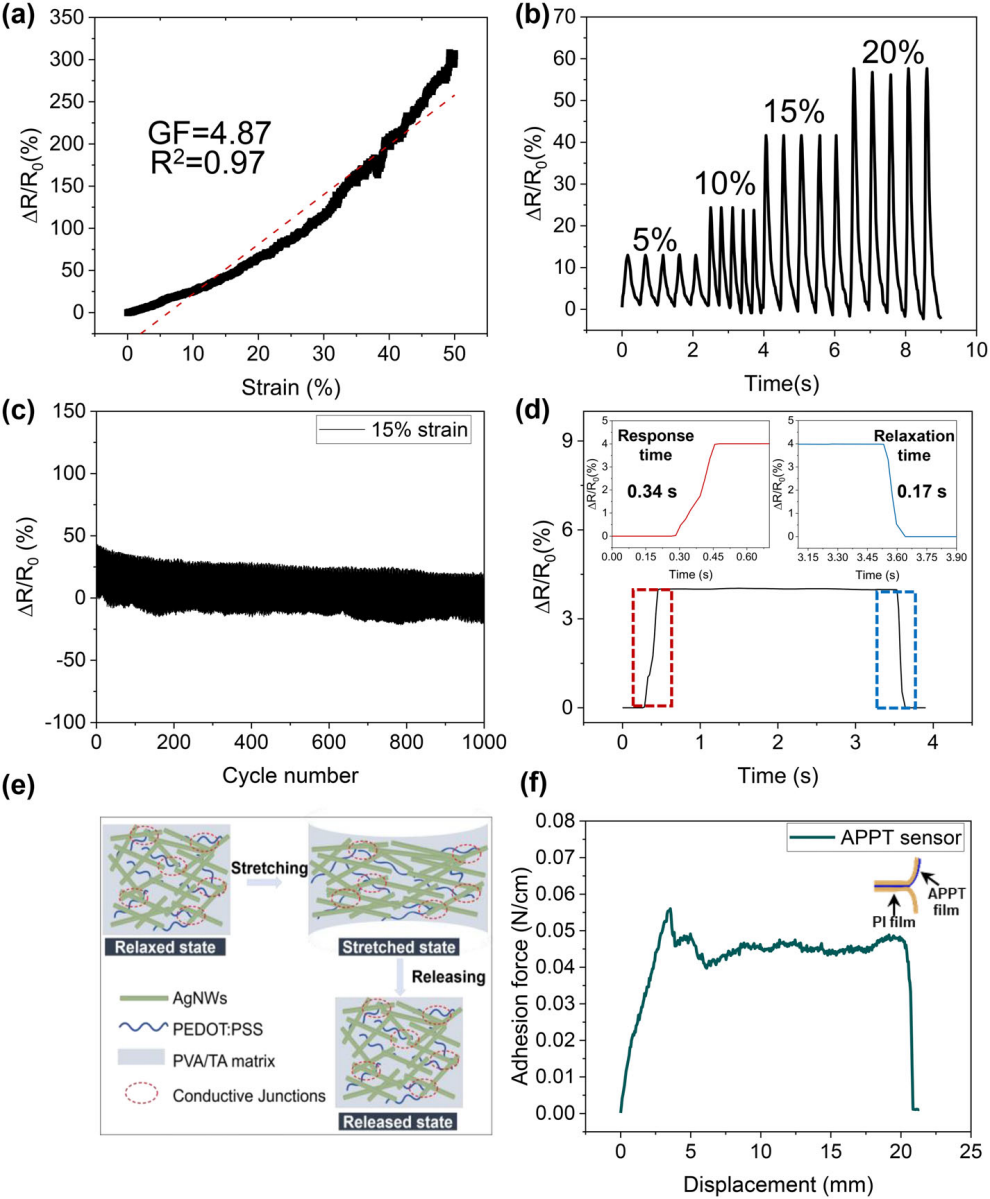
Characterization of APPT sensors. Relative resistance changes (ΔR/R_0_) of the APPT sensor under (a) monotonic tensile strains, (b) five cyclic stretching‐releasing loadings with peak strains from 5% to 20%, and (c) 1000 repeated stretching‐releasing cycles at 15% strain. (d) Response and relaxation times of the APPT sensor when subjected to 2% strain at 50%/s. (e) Schematic illustration of the strain‐sensing mechanism. (f) 180° peel force per unit width of the APPT sensor.

In addition, the interfacial adhesion of the sensor was measured using a 180° peeling test, yielding a maximum peel strength of approximately 0.056 N cm^−^^1^ (Figure 7e). Finally, the experimentally measured properties were compared with the ANN–GA predicted results (Figure S13). The deviations between experimental and predicted values are within 13%, confirming the reliability of the ANN–GA modeling and optimization strategy [46]. Overall, as summarized in Table S2, the APPT sensor is distinct from many reported self‐adhesive sensors that rely on hydrated gel matrices or multilayer structures. The APPT film maintains intrinsic dry adhesion together with moderate sensitivity, stretchability, and cyclic stability, supporting reliable sensing performance.

### 2.7 Applications Demonstration of APPT Sensors

The optimized APPT sensor's potential in real‐time motion sensing and human‐machine interfaces was examined in this section. As shown in Figure 8a, the APPT sensor successfully detected swallowing‐induced local skin mechanical deformation when mounted on the throat. Similarly, the local abdominal skin deformation associated with respiration was successfully recorded by attaching the sensor to an elastic band positioned around the abdomen. As shown in Figure 8b, periodic resistance variations corresponding to inhalation and exhalation cycles were clearly resolved, demonstrating the capability of the sensor to capture physiological motions. Moreover, finger joint bending was evaluated by attaching the APPT sensor to the index finger joint. As shown in Figure 8c, distinct relative resistance changes (ΔR/R_0_) of approximately 0.21, 0.44, and 0.65 were observed at bending angles of 30°, 60°, and 90°, respectively, corresponding to strains of ∼14%, 25%, and 37%. These results indicate that the APPT sensor can reliably distinguish different joint bending angles with high sensitivity and rapid response.

**FIGURE 8:**
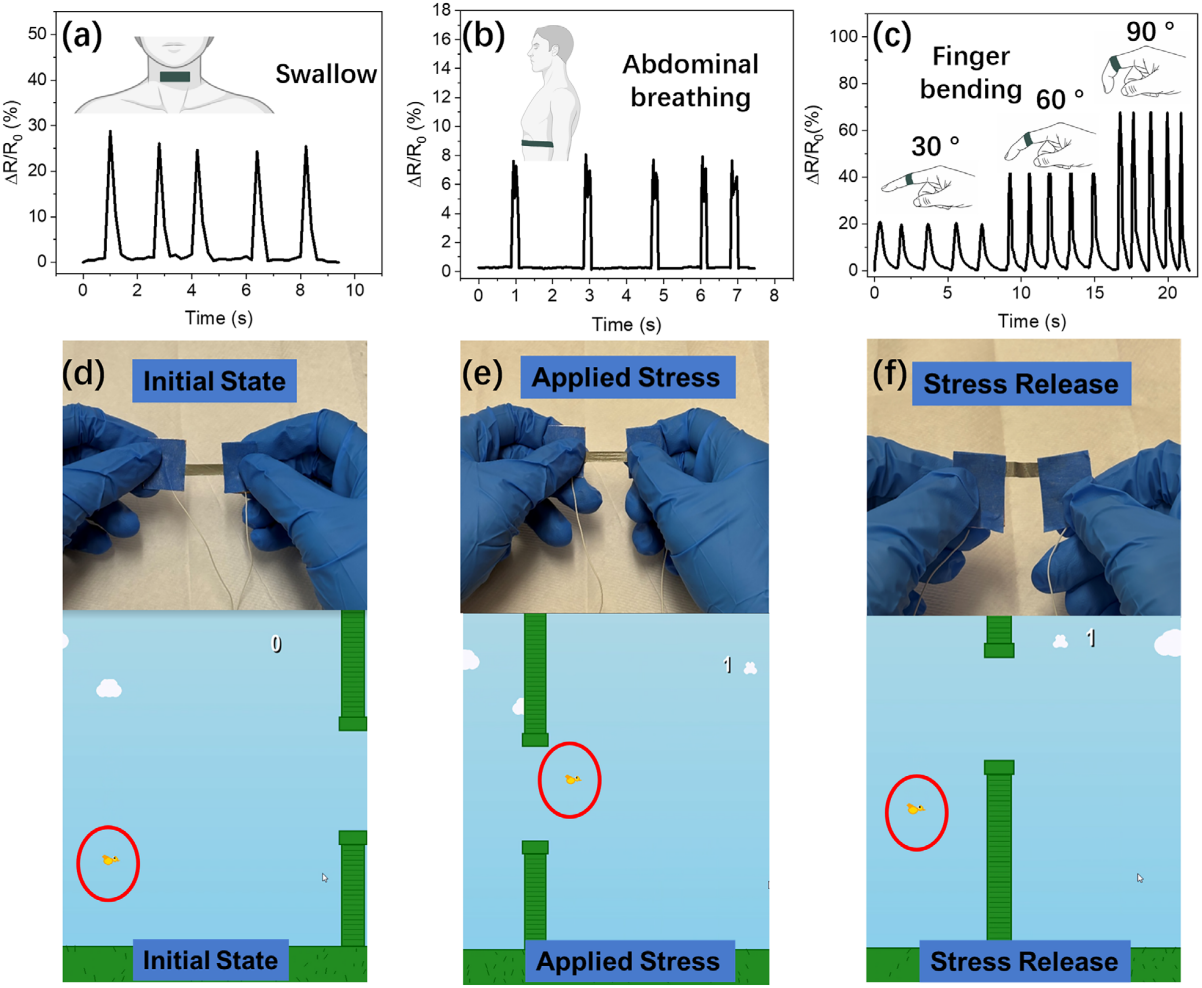
Application of the APPT sensors in human motion detection and the human–machine interfaces. Relative resistance changes of the APPT sensor induced by (a) throat swallowing motion; (b) abdominal respiration; (c) finger joint bending. Demonstration of a human–machine interface based on the APPT sensor using a Flappy Bird game, showing (d) the initial state, (e) applying stress, and (f) releasing stress, where the sensor output is mapped to real‐time control of the game character.

Furthermore, the potential of the APPT sensor for human‐machine interface application was demonstrated in a Flappy Bird game. As shown in Figure 8d–f and Video S3, the resistance change of an APPT sensor was converted into a control signal to drive the vertical motion of the game character. In the absence of external stress, the character moved downward under gravity, whereas applying stress drove upward motion, and releasing the stress caused it to descend again. This demonstration confirms the capability of the APPT sensor to function as an effective input interface for interactive and entertainment systems.

In summary, these results demonstrate that the APPT sensor enables accurate detection of diverse human motions and supports real‐time signal transduction for human‐machine interaction, underscoring its strong potential for wearable sensing and human‐machine interface applications.

## 3 Conclusions

In this work, a data‐driven optimization strategy combining ANN and GA was developed to guide the design of self‐adhesive conductive polymer composites for wearable applications. This approach captures the combined effects of multiple components and identifies compositions that balance mechanical and electrical performance. Compared with conventional trial‐and‐error methods, it enables more efficient exploration of the compositional space. By tailoring the optimization objectives, stretchable epidermal electrodes and strain sensors were developed using a unified material platform.

For epidermal electrode applications, the optimized APPT electrode exhibits a large elongation at break of approximately 177% and maintains a low skin‐electrode contact impedance (∼ 72 kΩ· at 10 Hz), supporting conformal and stable skin contact. In this optimal composition, the PVA matrix provides mechanical flexibility for large elongation, while TA improves adhesion to skin. The combined conductive network of PEDOT:PSS and AgNWs maintains stable electrical pathways, leading to low skin‐electrode contact impedance. Systematic EMG, ECG, EOG, and EEG measurements demonstrate the electrode delivers signal quality comparable to commercial Ag/AgCl gel electrodes, while offering superior performance under wet‐skin conditions and prolonged wear. The sustained signal stability is closely associated with the intrinsic self‐adhesive property and conformal skin contact of the APPT electrode. Moreover, the optimized APPT sensor shows large stretchability (up to 153%), stable and reversible piezoresistive behavior with a gauge factor of approximately 4.87, fast dynamic response, and excellent cyclic durability. In this optimal composition, a better balance between the PVA matrix and the conductive PEDOT:PSS/AgNWs phase maintains stretchability and enables larger resistance changes under strain, leading to higher sensitivity. Practical demonstrations, including swallowing detection, respiration monitoring, finger joint bending sensing, and human‐machine interface control, confirm the versatility and reliability of the APPT sensor for wearable sensing. This work highlights the effectiveness of ANN‐GA‐guided optimization in enabling application‐specific material design within a shared compositional framework. The demonstrated APPT electrodes and sensors provide a promising platform for next‐generation wearable bioelectronics, offering stable long‐term performance, simplified skin interfacing without conductive gels, and broad applicability in physiological monitoring and human‐machine interaction.

## 4 Experimental Section

### 4.1 Materials

PEDOT:PSS, PVA (Mw ≈ 89 000), polyvinylpyrrolidone (PVP, Mw ≈ 40 000), AgNO_3_ powder, and sodium chloride (NaCl) powder were purchased from Sigma–Aldrich. Glycerol and tannic acid were obtained from Chem Supply.

### 4.2 Synthesis of AgNWs

AgNWs were synthesized according to a previously published polyol method [54, 55]. First, 5.86 g of PVP was dissolved into 190 mL of glycerol and heated to 90°C. Subsequently, 1.58 g of AgNO_3_ was added, followed by the introduction of a premixed solution containing 10 mL of glycerol, 59 mg of NaCl, and 0.5 mL of H_2_O. The mixture was then heated up to 200°C and maintained under gentle stirring (50 rpm) until the solution gradually turned gray‐green, indicating the formation of AgNWs. Afterward, the reaction was stopped by transferring the hot reaction mixture into a beaker containing cold Milli‐Q water. The solution was left overnight, after which the supernatant containing silver nanoparticles and PVP was removed. This purification procedure was repeated three times to obtain the final aqueous AgNWs dispersion.

### 4.3 Fabrication Processes of APPT Films

The AgNWs solution was mixed with PEDOT:PSS solution, PVA solution (5 wt.%), and TA powder, followed by stirring for 30 min at room temperature. The APPT films were prepared by solution‐casting the above blending solution into a mold (25 mm × 25 mm) and dried at 60°C for at least 2 h. Finally, the resultant APPT films were peeled off after cooling down.

### 4.4 Characterization

Conductivity measurements of all samples were carried out using the 4‐point probe method (M‐3 type four‐probe tester, Suzhou Jingge Electronic Co., Ltd). A square film of APPT composite (25 mm of length, 25 mm of width) was used to test the sample electrical conductivity. The thickness of the sample film was obtained using a digital vernier caliper. The electrode‐skin interfacial impedance was measured with the dual‐electrode method using an LCR meter (MFIA, Zurich Instruments) over a frequency range from 1 to 10^4^ Hz at a voltage of 1 V. The tensile measurements were conducted using a universal testing machine (Instron Model 5567 testing system, USA). The uniaxial strain was applied at a ramp rate of 10 mm/min. The electromechanical properties of the APPT films were analyzed by measuring the change in resistance using a digital multimeter (Keysight E4980 AL) when subjected to a tensile strain. The response‐relaxation time was characterized by applying 2% strain at a strain rate of 50%/s and holding for 3 s before releasing the strain at the same strain rate. The Adhesion forces of the APPT films adhering to various substrates were measured using an 180° peeling test with the universal testing machine. All substrates were treated using 3 m tape to remove surface contaminants. Then, a free‐standing APPT film was attached to the diverse substrates. The dimensions of the samples were 5 mm (width) and 25 mm (length). Loading‐displacement curves were collected at a peeling test speed of 20 mm/min. The adhesion strength was determined by averaging the peeling force (F), followed by dividing it with the width of the sample (w).

### 4.5 Biopotential Measurement

The EMG signals were measured using the APPT electrodes as the electrodes. Two conductive electrodes (2 cm × 1 cm) were mounted on the forearm, and the distance between the two electrodes was 1 cm. Then a certain force was applied to the grip meter by hand to stimulate the generation of electromyographic signals. To record ECG signals, two electrodes were placed on each forearm, and one reference electrode on the back of the hand. To record EOG signals, two electrodes were placed on both sides of the temples, and one reference electrode behind the earlobe. To record EEG signals, one electrode was placed on the forehead and one reference electrode behind the earlobe. All the signals were recorded by a PSG system (Compumedics Grael 4K). All the recorded signals were processed with a bandpass filter (0.5–150 Hz). For comparison between APPT electrodes and commercial electrodes, the commercial Ag/AgCl electrode was also used following the same procedure.

Human‐machine interface application setup: The Flappy Bird Game was done in Python. The game principle is that the bird flies; its longitude is controlled depending on the applied stress, and the bird passes through the walls. Also, gravity is applied in the game. The APPT sensor signal acquired from the Arduino Uno platform was received by the computer and used for game control (Figure S14).

## Author Contributions

**Chun Hui Wang**: conceptualization, methodology, funding acquisition, Writing – review and editing. **Milad Razbin**: methodology, validation, software. **Wenlong Cheng**: writing – review and editing, conceptualization, methodology, supervision. **Wei Chen**: conceptualization, methodology, writing – review and editing, funding acquisition. **Chen Sang**: methodology, software. **Markus Müllner**: writing – review and editing, resources, funding acquisition, conceptualization, methodology. **Shilei Wang**: methodology, software. **Shuying Wu**: conceptualization, methodology, supervision, funding acquisition, project administration, resources, writing – review and editing. **Xuan Li**: conceptualization, methodology, software, data curation, investigation, validation, formal analysis, writing – original draft, writing – review and editing. **Shuhua Peng**: conceptualization, methodology, writing – review and editing. **Danish Tahir**: methodology, software.

## Ethical Approval

These experiments were conducted in accordance with the National Statement on Ethical Conduct in Human Research and the Australian Code for the Responsible Conduct of Research. Ethical approval was obtained from the University of Sydney Human Research Ethics Committee under protocol number HE001668.

## Conflicts of Interest

The authors declare no conflicts of interest.

## Supporting information




**Supporting File 1**: advs76928‐sup‐0001‐SuppMat.docx.

**Supporting File 2**: advs76928‐sup‐0002‐VideoS1.mp4.

**Supporting File 3**: advs76928‐sup‐0003‐VideoS2.mp4.

**Supporting File 4**: advs76928‐sup‐0004‐VideoS3.mp4.

## Data Availability

The data that support the findings of this study are available from the corresponding author upon reasonable request.

## References

[1] F. Zhang , S. Wu , S. Peng , Z. Sha , and C. H. Wang , “Synergism Of Binary Carbon Nanofibres And Graphene Nanoplates In Improving Sensitivity And Stability Of Stretchable Strain Sensors,” Composites Science and Technology 172 (2019): 7–16, 10.1016/j.compscitech.2018.12.031.

[2] S. Wu , R. B. Ladani , J. Zhang , et al., “Aligning Multilayer Graphene Flakes With An External Electric Field To Improve Multifunctional Properties Of Epoxy Nanocomposites,” Carbon 94 (2015): 607–618, 10.1016/j.carbon.2015.07.026.

[3] L. Yin , Y. Wang , J. Zhan , et al., “Chest‐Scale Self‐Compensated Epidermal Electronics For Standard 6‐Precordial‐Lead ECG,” npj Flexible Electronics 6 (2022): 29, 10.1038/s41528-022-00159-7.

[4] S. Mirjalali , S. Peng , Z. Fang , C.‐H. Wang , and S. Wu , “Wearable Sensors for Remote Health Monitoring: Potential Applications for Early Diagnosis of Covid‐19,” Advanced Materials Technologies 7 (2022): 2100545, 10.1002/admt.202100545. PMC8646515 34901382

[5] S. Wu , J. Zhang , R. B. Ladani , et al., “Novel Electrically Conductive Porous PDMS/Carbon Nanofiber Composites for Deformable Strain Sensors and Conductors,” ACS Applied Materials & Interfaces 9 (2017): 14207–14215, 10.1021/acsami.7b00847. 28398032

[6] J.‐W. Seo , H. Kim , K. Kim , S. Q. Choi , and H. J. Lee , “Calcium‐Modified Silk as a Biocompatible and Strong Adhesive for Epidermal Electronics,” Advanced Functional Materials 28 (2018): 1800802, 10.1002/adfm.201800802.

[7] Z. Wang , X. Xu , Y. Xu , W. Lin , and Z. Peng , “A Ternary Heterogeneous Hydrogel With Strength Elements For Resilient, Self‐Healing, And Recyclable Epidermal Electronics,” npj Flexible Electronics 6 (2022): 51, 10.1038/s41528-022-00175-7.

[8] A. Y. Hannun , P. Rajpurkar , M. Haghpanahi , et al., “Cardiologist‐Level Arrhythmia Detection And Classification In Ambulatory Electrocardiograms Using A Deep Neural Network,” Nature Medicine 25 (2019): 65–69, 10.1038/s41591-018-0268-3. PMC6784839 30617320

[9] K. Morozumi , T. Fujiwara , T. Endou , et al., “Quantitative and Qualitative Evaluation of Knee Electromyograms by a Bluetooth‐communication Gait Analyzer: Integration and Power Spectral Analysis of Surface Electromyograms,” Journal of Physical Therapy Science 22 (2010): 259–265, 10.1589/jpts.22.259.

[10] S. Peng , S. Wu , Y. Yu , B. Xia , N. H. Lovell , and C. H. Wang , “Multimodal Capacitive and Piezoresistive Sensor for Simultaneous Measurement of Multiple Forces,” ACS Applied Materials & Interfaces 12 (2020): 22179–22190, 10.1021/acsami.0c04448. 32302480

[11] S. Kabiri Ameri , R. Ho , H. Jang , et al., “Graphene Electronic Tattoo Sensors,” ACS Nano 11 (2017): 7634–7641, 10.1021/acsnano.7b02182. 28719739

[12] N. D. Truong , A. D. Nguyen , L. Kuhlmann , et al., “Convolutional Neural Networks For Seizure Prediction Using Intracranial And Scalp Electroencephalogram,” Neural Networks 105 (2018): 104–111, 10.1016/j.neunet.2018.04.018. 29793128

[13] P. Zhu , Y. Zhang , A. Wu , et al., “A Damping And Adhesive Hydrogel Electrode For Continuous High‐Fidelity Dynamic Electrophysiological Monitoring And Human–Machine Interaction,” Nano Research 19 (2026): 94908565.

[14] B. Chen , R. Yu , J. Wang , et al., “Biomaterials‐Based Hydrogel with Superior Bio‐Mimetic Ionic Conductivity and Tissue‐Matching Softness for Bioelectronics,” Advanced Functional Materials 36 (2026): 27495.

[15] N. Alsaafeen , I. Ziogas , S. Alsaedi , et al., “Systematic Benchmarking of a Noise‐Tolerant Conductive Hydrogel Electrode for Epidermal Bioelectronics,” Advanced Science 13 (2026): 15131. 10.1002/advs.202515131 PMC13067809 41262003

[16] M. Xiao , X. Zhang , Y. Luo , R. Xie , K. Tao , and J. Wu , “Engineering Hydrogel‐Based Conformal Epidermal Electrodes For Human‐Machine Interaction,” Soft Science 5 (2025): 40.

[17] S. K. Sinha , Y. Noh , N. Reljin , et al., “Screen‐Printed PEDOT:PSS Electrodes on Commercial Finished Textiles for Electrocardiography,” ACS Applied Materials & Interfaces 9 (2017): 37524–37528, 10.1021/acsami.7b09954. 29020777

[18] A. Zucca , C. Cipriani , S. T. Sudha , D. Ricci , V. Mattoli , and F. Greco , “Conformable Electronics: Tattoo Conductive Polymer Nanosheets for Skin‐Contact Applications,” Advanced Healthcare Materials 4 (2015): 941, 10.1002/adhm.201570039. 25702914

[19] S. Peng , Y. Yu , S. Wu , and C.‐H. Wang , “Conductive Polymer Nanocomposites for Stretchable Electronics: Material Selection, Design, and Applications,” ACS Applied Materials & Interfaces 13 (2021): 43831–43854, 10.1021/acsami.1c15014. 34515471

[20] C. Yang , D. Zhang , D. Wang , X. Chen , and H. Luan , “Ultra‐Sensitive, Stretchable, And Bidirectional Wearable Strain Sensor For Human Motion Detection,” Journal of Materials Chemistry C 10 (2022): 7076–7086, 10.1039/D2TC00735E.

[21] H. Zheng , N. Lin , Y. He , and B. Zuo , “Self‐Healing, Self‐Adhesive Silk Fibroin Conductive Hydrogel as a Flexible Strain Sensor,” ACS Applied Materials & Interfaces 13 (2021): 40013–40031, 10.1021/acsami.1c08395. 34375080

[22] A. Chananipoor , Z. Azizi , B. Raei , and N. Tahmasebi , “Optimization Of The Thermal Performance Of Nano‐Encapsulated Phase Change Material Slurry In Double Pipe Heat Exchanger: Design Of Experiments Using Response Surface Methodology (RSM),” Journal of Building Engineering 34 (2021): 101929, 10.1016/j.jobe.2020.101929.

[23] L. Ran , G. Yan , V. Goyal , et al., “Advancing Solar Thermal Utilization By Optimization Of Phase Change Material Thermal Storage Systems: A Hybrid Approach Of Artificial Neural Network (ANN)/Genetic Algorithm (GA),” Case Studies in Thermal Engineering 64 (2024): 105513, 10.1016/j.csite.2024.105513.

[24] K. Liu , R. Zhang , S. Zhang , et al., “Composition Design Of High‐Performance Copper Alloy By Coupling Artificial Neural Network And Genetic Algorithm,” Computational Materials Science 229 (2023): 112449, 10.1016/j.commatsci.2023.112449.

[25] K. Venkateshwar , S. H. Tasnim , S. A. Gadsden , and S. Mahmud , “Artificial Neural Network‐Genetic Algorithm Optimized Graded Metal Foam,” Journal of Energy Storage 51 (2022): 104386, 10.1016/j.est.2022.104386.

[26] J. Li , A. D. Celiz , J. Yang , et al., “Tough Adhesives For Diverse Wet Surfaces,” Science 357 (2017): 378–381, 10.1126/science.aah6362. PMC5905340 28751604

[27] E. Noh , H. K. Um , H. J. Park , et al., “Stretchable Dry Adhesives for Robust Epidermal Biosignal Sensing,” Advanced Healthcare Materials 14 (2025): 00878, 10.1002/adhm.202500878. 40702837

[28] Y. Liu , Y. Cheng , L. Shi , R. Wang , and J. Sun , “Breathable, Self‐Adhesive Dry Electrodes for Stable Electrophysiological Signal Monitoring During Exercise,” ACS Applied Materials & Interfaces 14 (2022): 12812–12823, 10.1021/acsami.1c23322. 35234456

[29] J. X. M. Chen , T. Chen , Y. Zhang , et al., “Conductive Bio‐based Hydrogel for Wearable Electrodes via Direct Ink Writing on Skin,” Advanced Functional Materials 34 (2024): 2403721.

[30] S. Song , L. Qi , C. Lang , et al., “Self‐Adhesive And Breathable Nanofibrous Dry Patterned Electrode For Reliable ECG Monitoring,” Applied Materials Today 48 (2026): 102999.

[31] X. Niu , X. Gao , T. Wang , W. Wang , and H. Liu , “Ordered Nanopillar Arrays of Low Dynamic Noise Dry Bioelectrodes for Electrocardiogram Surface Monitoring,” ACS Applied Materials & Interfaces 14 (2022): 33861–33870, 10.1021/acsami.2c08318. 35830904

[32] J. Wang , A. Karimipour , S. M. Sajadi , et al., “Analysis of the Non‐Newtonian Behavior and Viscosity of GNSs‐CuO/Liquid EG Hybrid Nanofluid: An Experimental and Feed‐Forward ANN Study,” International Journal of Thermophysics 44 (2023): 103, 10.1007/s10765-023-03196-0.

[33] O. Chaabouni and S. Boufi , “Cellulose Nanofibrils/Polyvinyl Acetate Nanocomposite Adhesives With Improved Mechanical Properties,” Carbohydrate Polymers 156 (2017): 64–70, 10.1016/j.carbpol.2016.09.016. 27842853

[34] K. Angela , S. Taddeo , and M. James , “Predicting Global Solar Radiation Using an Artificial Neural Network Single‐Parameter Model,” Advances in Artificial Neural Systems 2011 (2011): 751908, 10.1155/2011/751908.

[35] M. Razbin , M. Vahdani , S. A. Moshizi , et al., “Application Of Soft Computing Techniques In The Optimization Of 3D‐Printed Piezoresistive Sensors,” Sensors and Actuators A: Physical 385 (2025): 116277, 10.1016/j.sna.2025.116277.

[36] M. Sohrabi , M. Razbin , M. Pourtavvaf , R. Bagherzadeh , and M. M. Mirmahale , “Exercising Hybrid Model To Design An Optimized Electrospun Polyamide‐6 Nanofibrous Mat For Air Filtration Applications,” The Journal of The Textile Institute 114 (2023): 1667–1681, 10.1080/00405000.2022.2145440.

[37] W. Li , R. Wang , T. Zhang , M. Ming , and K. Li , “Reinvestigation Of Evolutionary Many‐Objective Optimization: Focus On The Pareto Knee Front,” Information Sciences 522 (2020): 193–213, 10.1016/j.ins.2020.03.007.

[38] A. Setämaa‐Kärkkäinen , K. Miettinen , and J. Vuori , “Best Compromise Solution For A New Multiobjective Scheduling Problem,” Computers & Operations Research 33 (2006): 2353–2368, 10.1016/j.cor.2005.02.006.

[39] O. Cuate and O. Schütze , Mathematics 8, (2020): 1651.

[40] R. V. Rao and R. J. Lakshmi , “Ranking Of Pareto‐Optimal Solutions And Selecting The Best Solution In Multi‐ And Many‐Objective Optimization Problems Using R‐Method,” Soft Computing Letters 3 (2021): 100015, 10.1016/j.socl.2021.100015.

[41] L. Belhoul , L. Galand , and D. Vanderpooten , “An Efficient Procedure For Finding Best Compromise Solutions To The Multi‐Objective Assignment Problem,” Computers & Operations Research 49 (2014): 97–106, 10.1016/j.cor.2014.03.016.

[42] E. H. Kim , H. Han , S. Yu , et al., “Interactive Skin Display With Epidermal Stimuli Electrode,” Advanced Science 6 (2019): 1802351, 10.1002/advs.201802351. PMC6662062 31380180

[43] J. Y. Oh , S. Rondeau‐Gagné , Y.‐C. Chiu , et al., “Intrinsically Stretchable And Healable Semiconducting Polymer For Organic Transistors,” Nature 539 (2016): 411–415, 10.1038/nature20102. 27853213

[44] J. J. Kim , Y. Wang , H. Wang , S. Lee , T. Yokota , and T. Someya , “Skin Electronics: Next‐Generation Device Platform for Virtual and Augmented Reality,” Advanced Functional Materials 31 (2021): 2009602, 10.1002/adfm.202009602.

[45] A. M. Nardes , M. Kemerink , R. A. J. Janssen , et al., “Microscopic Understanding of the Anisotropic Conductivity of PEDOT:PSS Thin Films,” Advanced Materials 19 (2007): 1196–1200, 10.1002/adma.200602575.

[46] P. Rong , Y. Zuo , J. Lin , et al., “In Situ Stress Prediction Model In Complex Geology: A Hybrid GA‐ANN With Nonlinear Boundary Condition,” Journal of Rock Mechanics and Geotechnical Engineering 17 (2025): 4349–4366, 10.1016/j.jrmge.2024.11.003.

[47] H. Kim , E. Kim , C. Choi , and W.‐H. Yeo , “Advances in Soft and Dry Electrodes for Wearable Health Monitoring Devices,” Micromachines 13, no. 4 (2022): 629. 10.3390/mi13040629 PMC9029742 35457934

[48] Y. Xiao , M. Wang , Y. Li , et al., “High‐Adhesive Flexible Electrodes and Their Manufacture: A Review,” Micromachines 12, (2021): 1505. 10.3390/mi12121505 PMC8704330 34945355

[49] S. Seyedin , P. Zhang , M. Naebe , et al., “Textile Strain Sensors: A Review Of The Fabrication Technologies, Performance Evaluation And Applications,” Materials Horizons 6 (2019): 219–249, 10.1039/C8MH01062E.

[50] J. Feng , “Multi‐Layered Crack‐Like Flexible Strain Sensor with Gradient Concentration Structure Based on Mxene‐Ta‐Pva,” Theoretical and Natural Science 104 (2025): 48–54.

[51] D. Tahir , X. Li , M. Razbin , et al., “Eco‐Friendly, Flexible And Stretchable Printed Electronics Based On A Sustainable Elastic Substrate And Ink,” Journal of Materials Chemistry A 13 (2025): 34393–34408, 10.1039/D5TA06546A.

[52] J. H. Lee , V. Raman , C. Kang , H. U. Ha , H. J. Seok , and H. K. Kim , “Highly Stretchable Transparent Bar Coated Ag NW/PEDOT:PSS Hybrid Electrode For Wearable And Stretchable Devices,” RSC Advances 12 (2022): 3055–3061, 10.1039/D1RA08173J. PMC8979090 35425295

[53] Y. N. Chen , L. Peng , T. Liu , Y. Wang , S. Shi , and H. Wang , “Poly(vinyl alcohol)–Tannic Acid Hydrogels With Excellent Mechanical Properties and Shape Memory Behaviors,” ACS Applied Materials & Interfaces 8 (2016): 27199–27206, 10.1021/acsami.6b08374. 27648478

[54] S. Peng , S. Wu , Y. Yu , P. Blanloeuil , and C. H. Wang , “Nano‐Toughening Of Transparent Wearable Sensors With High Sensitivity And A Wide Linear Sensing Range,” Journal of Materials Chemistry A 8 (2020): 20531–20542, 10.1039/D0TA05129B.

[55] S. Wu , S. Peng , Y. Yu , and C.‐H. Wang , “Strategies for Designing Stretchable Strain Sensors and Conductors,” Advanced Materials Technologies 5 (2020): 1900908, 10.1002/admt.201900908.

