# Supplementary material for: Data‐Driven Design of Self‐Adhesive Epidermal Electrodes and Sensors
Source: Adv Sci (Weinh). 2026 Jul 31;13(60):e76928. doi: 10.1002/advs.76928 (PMC13426098; doi:10.1002/advs.76928)
Supplement: Supplementary file 1 [file ADVS-13-e76928-s002.docx]

**Data-Driven Design of Self-Adhesive Epidermal Electrodes and Sensors**

Xuan Li^a^, Shilei Wang^b^, Milad Razbin^a^, Danish Tahir^a^, Chen Sang^a^, Shuhua Peng ^c^, Markus Müllner ^de^, Wenlong Cheng^b^, Wei Chen^b^, Chun Hui Wang^c^, Shuying Wu^ae^*

^a^School of Aerospace, Mechanical and Mechatronic Engineering, The University of Sydney, Sydney, NSW 2006, Australia

^b^School of Biomedical Engineering, The University of Sydney, Sydney, NSW 2006, Australia

^c^School of Mechanical and Manufacturing Engineering, The University of New South Wales, Sydney, NSW 2052, Australia

^d^Key Centre for Polymers & Colloids, School of Chemistry, The University of Sydney, Sydney, NSW 2006, Australia

^e^The University of Sydney Nano Institute (Sydney Nano), The University of Sydney, Sydney, NSW 2006, Australia

*Corresponding author, Email: shuying.wu@sydney.edu.au

**Keywords**: Epidermal electrodes, Self-adhesive, Biopotential, Data-driven

**Table S1**. List of recently reported self-adhesive epidermal electrodes created based on different adhesion mechanisms and material systems, as well as their properties.

| **Adhesion mechanism** | **Materials** | **Conductivity** | **Skin-electrode Impedance** | **Adhesion** | **Elongation at the break** | **Ref. #** |
| --- | --- | --- | --- | --- | --- | --- |
| Hydrogen bonding | PEDOT:PSS/Ethylene Glycol/Zonyl FS-300/TA/D-sorbitol  Bottom layer: TA / D-sorbitol | < 180 Ω/sq | N/A | 0.87 N / cm (180° peel test on stainless steel plates) | ~ 50 % | 1 |
| Hydrophobic anchoring | AgNWs/α-CD/C9-PVA/Glycerol | N/A | 1217 kΩ at 10 HZ | 6.33 kPa （T-peel test on pig skin） | ~ 598% | 2 |
| Hydrogen bonding | CMC/PDA/ PEDOT:PSS / Phytic acid | 0.26 S/ m | N/A | 93.95 J /m²（lab shear on pig skin） | ~ 90 % | 3 |
| Hydrogen bonding | PVA Borax/ PEDOT:PSS | 2 × 10^-5^ S/m -5 × 10^-5^ S/m | 185 kΩ at 10 HZ | 1.57 N / cm^2^ (Perpendicular pull-off test on pig skin) | ~ 10000 % | 4 |
| Ultrathin structure and van der Waals | AgNWs/TPU/PDMS | N/A | 504 KΩ at 0.1 HZ | 243.23 ± 5.74 μJ / cm² (Plate separation test) | ~ 90 % | 5 |
| Micro/nanopillar interlocking | PANI/TPU | 37.6 S/m | 375.5 kΩ at 0.1 HZ | 69.6 mN/ cm (Perpendicular peel test on simulated skin) | ~ 291.9 % | 6 |
| Hydrogen bonding and ultrathin structure | AgNWs/PEDOT:PSS/PVA/TA | 16.5 S/cm | 72 kΩ at 10 Hz | 0.14 N / cm (180° peel test on pig skin) | ~ 177% | This work |

**Table S2**. List of recently reported self-adhesive sensors created based on different adhesion mechanisms and material systems, as well as their properties.

| **Adhesion mechanism** | **Materials** | **Adhesion** | **Elongation at the break** | **Gauge factor** | **Stability** | **Ref. #** |
| --- | --- | --- | --- | --- | --- | --- |
| Hydrogen bonding | AgMPs/WPU/glycerol/D-sorbitol | 7.91 N /m (90° peel test on pig kin) | ~ 874% | 103.01 (14.8–19.2 % strain);  8.48 (0–7.3 % strain);  GF=29.83 (7.3–14.8 % strain) | 1000 cycles at 5% strain | 7 |
| Hydrogen bonding | PEDOT:PSS/PVA/borax/glycerol | 0.10N /cm² (pull-off test on pigskin) | ~ 860% | 0.7 (0–300 % strain); 1.5 (300–600 % strain); 2.3 (600–866 % strain) | 1000 cycles at 100% | 8 |
| Hydrogen bonding | (R)−12-hydroxyethylhydrazine stearate (HSAH)/poly (N-hydroxyethyl acrylamide) | 105 kPa (pull-off test on pig skin) | ~ 500% | 2.5 (0-150 % strain) | 3000 cycles at 50% | 9 |
| Hydrophobic anchoring and van der Waals | Thioctic acid/ polymerizable hydrophobic /ionic liquid | 0.12 MPa (lap shear on pig skin) | ~ 1500% | 1.04 (< 600 % strain); 1.85 (> 600 % strain) | 1200 cycles at 100% strain | 10 |
| Hydrogen bonding | AgNWs/TPU/PDMS | 5.00 kPa  (lap shear on pig skin) | ~ 384% | 1.86 (0-200 % strain) | 100 cycles at 50%, 100% and 200% strain | 11 |
| Hydrogen bonding and ultrathin structure | AgNWs/PEDOT:PSS/PVA/TA | 0.056 N/cm  (180° peel test on PI) film) | ~ 153% | 4.79 (0-60 % strain) | 1000 cycles at 15 % | This work |

**Table S3**. Control parameters of four different components.

| Components | Range |
| --- | --- |
| PEDOT:PSS | 5 wt%-25 wt% |
| AgNWs | 5 wt%-50 wt% |
| PVA | 10 wt%-40 wt% |
| TA | 40 wt%-90 wt% |

*Constraint condition: PEDOT:PSS+AgNWs+PVA+TA = 100 % (weight fraction)

**Table S4**. Different permutations of control variables and their corresponding responses.

| Samples |  | Component 1 | Component 2 | Component 3 | Component 4 |  | Response parameter 1 | Response parameter 2 | Response parameter 3 | Response parameter 4 |  | Datasets | |
| --- | --- | --- | --- | --- | --- | --- | --- | --- | --- | --- | --- | --- | --- |
|  |  | PEDOT:PSS  (wt %) | AgNWs  (wt%) | PVA  (wt%) | TA  (wt%) |  | Conductivity  (S/cm) | Elongation  (%) | Adhesion  (N/cm) | Gauge  factor |  | Training | Testing |
| 1 |  | 21 | 5 | 34 | 40 |  | 18.40 | 64.00 | 0.03 | 0.22 |  | + | - |
| 2 |  | 17 | 5 | 25 | 53 |  | 15.88 | 260.00 | 0.04 | 0.25 |  | + | - |
| 3 |  | 6 | 33 | 11 | 51 |  | 23.36 | 34.00 | 0.03 | 2.09 |  | + | - |
| 4 |  | 5 | 45 | 10 | 40 |  | 126.17 | 12.00 | 0.02 | 0.19 |  | + | - |
| 5 |  | 12 | 36 | 10 | 42 |  | 29.91 | 25.00 | 0.03 | 2.76 |  | + | - |
| 6 |  | 7 | 33 | 20 | 40 |  | 24.58 | 250.00 | 0.03 | 10.50 |  | + | - |
| 7 |  | 10 | 6 | 40 | 44 |  | 5.38 | 231.00 | 0.02 | 0.40 |  | + | - |
| 8 |  | 5 | 5 | 34 | 56 |  | 4.33 | 361.00 | 0.05 | 1.23 |  | + | - |
| 9 |  | 5 | 18 | 37 | 40 |  | 3.34 | 369.00 | 0.01 | 0.92 |  | + | - |
| 10 |  | 7 | 6 | 18 | 69 |  | 3.25 | 171.00 | 0.11 | 1.57 |  | + | - |
| 11 |  | 19 | 18 | 11 | 52 |  | 23.91 | 25.10 | 0.04 | 0.45 |  | + | - |
| 12 |  | 20 | 23 | 17 | 40 |  | 24.53 | 23.50 | 0.04 | 0.36 |  | - | + |
| 13 |  | 25 | 25 | 10 | 40 |  | 27.47 | 20.90 | 0.03 | 0.59 |  | + | - |
| 14 |  | 6 | 18 | 24 | 53 |  | 7.13 | 250.00 | 0.07 | 0.25 |  | + | - |
| 15 |  | 12 | 8 | 10 | 70 |  | 8.66 | 77.00 | 0.09 | 0.23 |  | + | - |
| 16 |  | 5 | 5 | 10 | 80 |  | 2.50 | 209.00 | 0.30 | 0.63 |  | + | - |
| 17 |  | 23 | 5 | 10 | 62 |  | 27.54 | 21.30 | 0.02 | 0.92 |  | + | - |
| 18 |  | 5 | 15 | 11 | 69 |  | 3.63 | 220.00 | 0.14 | 0.55 |  | + | - |
| 19 |  | 25 | 10 | 19 | 46 |  | 22.29 | 33.00 | 0.03 | 0.61 |  | - | + |
| 20 |  | 12 | 16 | 31 | 41 |  | 27.23 | 184.00 | 0.03 | 0.34 |  | + | - |
| 21 |  | 12 | 5 | 20 | 63 |  | 4.82 | 114.00 | 0.10 | 0.43 |  | - | + |
| 22 |  | 15 | 20 | 10 | 55 |  | 22.67 | 11.60 | 0.04 | 0.87 |  | - | + |
| 23 |  | 5 | 5 | 40 | 50 |  | 3.56 | 315.00 | 0.03 | 0.13 |  | - | + |
| 24 |  | 5 | 17.5 | 25 | 52.5 |  | 6.57 | 147.00 | 0.15 | 1.32 |  | - | + |

(Response surface methodology is used to efficiently sample the high-dimensional compositional space with a limited number of experiments. Adhesion corresponds to the maximum peel strength obtained from the 180° peeling test. The gauge factor was calculated over the strain range of 0–50%. For samples exhibiting an elongation exceeding 50%, only the 0–50% strain region was used for gauge factor calculation; for samples with elongation below 50%, the gauge factor was calculated over the entire elongation range.)

**Figure S1**. Structural configuration of the neural network.

**Section S1.** Modeling details.

**Overview**: Artificial neural networks (ANNs) were employed to establish nonlinear relationships between material composition and electrode performance. The ANN framework was selected due to its strong capability in handling multivariable nonlinear systems.

**Data Preprocessing and Normalization**: Prior to model training, all experimental data were normalized to improve numerical stability, convergence efficiency, and generalization capability [12-13]. The normalization was performed using the following equation:

$X_{n}=\left( b-a \right)\frac{X{-X}_{min}}{X_{max}{-X}_{min}}+a$	Eq. (1)

where a and b represent the lower and upper bounds of normalization (0.1 and 0.9, respectively), x is the original data value, and $X_{min}$and $X_{max}$are the minimum and maximum values of the dataset. After optimization, the inverse transformation was applied to recover the original scale.

**Modeling details**: The ANN model adopted a feed-forward architecture with a single hidden layer. According to Kolmogorov’s theorem, a neural network with one hidden layer and a sufficient number of neurons is capable of approximating any continuous nonlinear function [14-15]. The mathematical formulation of a single-hidden-layer ANN can be expressed as:

$y=f^{\left( 2 \right)}\left( \sum_{j=1}^{m_{2}} w_{jk}^{2}f^{\left( 1 \right)}\left( \sum_{j=1}^{m_{1}} w_{ij}^{1}x_{i}+b_{j}^{1} \right)+b_{k}^{(2)} \right)$	Eq. (2)

where x denotes the input features, w represents the weight matrix, b signifies the bias terms, and f corresponds to the activation function. The symbols $m_{1}$ and $m_{2}$ specify the number of neurons in the input and hidden layers, respectively. In this study, a sigmoid tangent (tansig) activation function was employed in the hidden layer, while a linear activation function was used for the output layer [16]. The corresponding mathematical expressions are given as:

$tansig\left( y \right)=\frac{2}{(1+e^{-2y})}-1$	Eq. (3)

$Purelin\left( y \right)=y$	Eq. (4)

The ANN model was trained using the Levenberg–Marquardt backpropagation algorithm. Hyperparameters including the number of hidden neurons, learning rate, and number of training epochs were optimized using a grid search strategy. The optimal network configuration consisted of six hidden neurons, a learning rate of 0.6, and 1000 training epochs. The complete dataset comprising 20 experimental samples was randomly divided into training and testing subsets with a ratio of 18:2. In addition, k-fold cross-validation was applied to further evaluate the generalization capability and robustness of the ANN model.

The predictive performance of the ANN model was evaluated using the coefficient of determination (R²), mean squared error (MSE), and the total goodness function (TGF). The expressions of these evaluation metrics are given below:

$TGF=\frac{1}{N}\sum_{i=1}^{2} n_{i}(R_{i}^{2}+e^{{-MSE}_{i}})$	Eq. (5)

$R^{2}=1-\frac{\sum_{1}^{n} {(a_{i}-p_{i})}^{2}}{\sum_{1}^{n} {(a_{i}-\bar{a})}^{2}}$	Eq. (6)

$MSE=\frac{\sum_{1}^{n} {(a_{i}-p_{i})}^{2}}{n}$	Eq. (7)

where aᵢ and pᵢ denote the experimental and predicted values, respectively, ā represents the average experimental value, and N is the total number of data points.

The total goodness function (TGF) integrates the effects of R², MSE, and the data split ratio to provide a comprehensive assessment of model performance. A TGF value approaching 2 indicates optimal predictive capability.

**Section S2.** Optimization details.

**Overview**: Genetic algorithm (GA) optimization was performed using trained artificial neural network (ANN) models as surrogate predictors of electrode performance. The ANN models were employed to rapidly evaluate electrical conductivity, elongation at break, adhesion, and gauge factor for arbitrary compositional inputs, thereby enabling efficient exploration of the design space without additional experimental measurements.

**Design variables:** The design variables were the compositional fractions of AgNWs, PEDOT:PSS, PVA, and TA, with the constraint that their sum equals unity.

**Fitness function formulation for APPT electrodes:** The optimization problem was formulated as a constrained maximization task. Electrical conductivity and interfacial adhesion were selected as the primary objectives and combined into a single fitness function after normalization to comparable scales. The normalized conductivity and adhesion values were summed to yield the fitness score for each candidate solution. Two additional performance metrics, elongation at break and gauge factor, were incorporated as inequality constraints. Specifically, the elongation at break was required to exceed 30% to ensure sufficient mechanical compliance for epidermal applications, while the gauge factor was constrained to remain below 2 to maintain signal stability. Candidate solutions violating either constraint were penalized and excluded from contributing positively to the fitness function.

**Fitness function formulation for APPT sensors:** For strain-sensing applications, the genetic algorithm framework and ANN models remained identical to those used for APPT electrode optimization. The optimization strategy was adapted by redefining the objective priorities to reflect the functional requirements of strain sensors. In this case, electrical conductivity was assigned a lower priority, while elongation at break, interfacial adhesion, and gauge factor were selected as the primary optimization objectives. Specifically, elongation at break was maximized to ensure large deformation capability, interfacial adhesion was maximized to maintain stable attachment during repeated stretching, and the gauge factor was optimized to achieve high strain sensitivity. Electrical conductivity was retained as a secondary constraint to ensure basic electrical continuity rather than being explicitly maximized. The selected objective formulation enables the identification of material compositions optimized for strain-sensing performance while maintaining sufficient electrical functionality.

**Genetic algorithm configuration**: Multi-objective optimization was performed using the MATLAB built-in gamultiobj function, which implements a non-dominated sorting genetic algorithm II (NSGA-II) framework [17-18]. A population size of 100 and a maximum of 100 generations were adopted. Tournament selection with a tournament size of 2 was used for parent selection. Two-point crossover was applied with a crossover fraction of 0.8. Mutation was performed using an adaptive feasible mutation operator with a mutation scale parameter of 0.2. Standard elitist non-dominated sorting and crowding-distance mechanisms were employed to maintain solution diversity and ensure convergence toward the Pareto front

**Extraction of optimal composition**: The composition corresponding to the maximum fitness value was selected as the optimal formulation and subsequently fabricated and experimentally characterized to validate the ANN–GA optimization results.

**Advantages and limitations of the ANN-GA framework:** Compared with conventional trial-and-error optimization, the ANN-GA framework enables model-guided and constrained multi-objective exploration of the compositional design space. In a conventional trial-and-error approach, a limited number of manually selected formulations are fabricated and evaluated individually, and the selected formulation is therefore restricted to the experimentally tested candidates. In contrast, the ANN establishes nonlinear composition-property relationships from the experimental dataset, while the GA searches the predefined feasible compositional domain to identify candidate formulations that balance competing performance requirements. This approach enables conductivity, elongation at break, adhesion, and gauge factor to be considered simultaneously under application-specific objectives and constraints.

However, several limitations should also be acknowledged. First, the predictive reliability of the ANN is inherently dependent on the size, quality, and compositional coverage of the experimental dataset. Therefore, the present model should only be applied within the predefined compositional ranges and the fabrication and characterization conditions represented by the current dataset; extrapolation to new material constituents or untested processing conditions is not warranted. Second, the ANN captures empirical composition-property relationships and does not, by itself, establish causal mechanisms. Third, the candidate solutions identified by the GA depend on the selected objective functions, constraints, and model uncertainty, and do not guarantee a global experimental optimum. Accordingly, the ANN–GA framework should be regarded as a decision-support tool for prioritizing candidate formulations, while fabrication and independent experimental validation remain essential.

**Table S5**. Six-fold cross-validation results for the conductivity ANN model.

| Fold | Training MSE | Training $R^{2}$ | Validation MSE | Validation $R^{2}$ |
| --- | --- | --- | --- | --- |
| 1 | 4.87E-05 | 0.998770974 | 0.007697726 | 0.961124994 |
| 2 | 0.000137 | 0.996528889 | 0.008382047 | 0.95766904 |
| 3 | 0.000132 | 0.99666307 | 0.001040343 | 0.994746064 |
| 4 | 4.06E-05 | 0.998975168 | 0.001386564 | 0.992997585 |
| 5 | 0.000151 | 0.996182262 | 0.001982977 | 0.989985582 |
| 6 | 2.8E-05 | 0.999292803 | 0.002759595 | 0.986063509 |

**Table S6**. Six-fold cross-validation results for the elongation at break ANN model.

| Fold | Training MSE | Training $R^{2}$ | Validation MSE | Validation $R^{2}$ |
| --- | --- | --- | --- | --- |
| 1 | 3.83569E-05 | 0.99910109 | 0.011745533 | 0.944947657 |
| 2 | 1.62246E-05 | 0.99961977 | 0.015999556 | 0.925008679 |
| 3 | 0.000158394 | 0.996287973 | 0.013381774 | 0.937278451 |
| 4 | 0.000125288 | 0.99706381 | 0.000627563 | 0.997058558 |
| 5 | 9.1281E-05 | 0.997860789 | 0.002045981 | 0.990410307 |
| 6 | 4.19008E-06 | 0.999901804 | 0.015999882 | 0.925007152 |

**Table S7**. Six-fold cross-validation results for the adhesion ANN model.

| Fold | Training MSE | Training $R^{2}$ | Validation MSE | Validation $R^{2}$ |
| --- | --- | --- | --- | --- |
| 1 | 0.00010438 | 0.999044335 | 0.006287602 | 0.988486554 |
| 2 | 0.000134725 | 0.998766499 | 0.001822957 | 0.99666192 |
| 3 | 4.34784E-05 | 0.999601926 | 0.009419248 | 0.982752088 |
| 4 | 0.00015612 | 0.998570613 | 0.002643784 | 0.995158874 |
| 5 | 0.000106276 | 0.999026974 | 0.004118653 | 0.992458191 |
| 6 | 0.000123906 | 0.998865559 | 0.024863325 | 0.954471901 |

**Table S8**. Six-fold cross-validation results for the gauge factor ANN model.

| Fold | Training MSE | Training $R^{2}$ | Validation MSE | Validation $R^{2}$ |
| --- | --- | --- | --- | --- |
| 1 | 4.85046E-07 | 0.999987968 | 0.010964997 | 0.945600518 |
| 2 | 8.09607E-06 | 0.999799169 | 0.00557469 | 0.972342881 |
| 3 | 0.000100044 | 0.997518308 | 0.006256767 | 0.968958961 |
| 4 | 0.000153537 | 0.996191356 | 0.009164433 | 0.954533467 |
| 5 | 5.12628E-05 | 0.998728376 | 0.008531594 | 0.957673105 |
| 6 | 9.93666E-05 | 0.997535115 | 0.011167876 | 0.944593995 |

**Figure S2**. Impedance spectrum of the APPT electrode in comparison with a commercial Ag/AgCl gel electrode measured in air.

**Figure S3**. Variations of the resistance of APPT electrode under tensile strain up to its failure strain.

**Figure S4**. Resistance response of the APPT electrode under repeated 1000 stretching–releasing cycles at 50% strain.

**Figure S5**. Photographs demonstrate the robust self-adhesive property of the APPT electrode on different substrates: (a) a glass slide (4.9 g), (b) a petri dish (9.3 g) and (c) a rubber mold (13.3 g).

**Figure S6**. Comparison between experimental and predicted values of conductivity, elongation, adhesion, and gauge factor for the APPT electrode.

**Figure S7.** (a) APPT electrodes and (b) Ag/AgCl gel electrodes during 9 h of wear under a gripping force of 5 kg for Participant 2.

**Figure S8.** (a) APPT electrodes and (b) Ag/AgCl gel electrodes during 9 h of wear under a gripping force of 5 kg for Participant 3.

**Figure S9**. 180° peeling force –displacement curves of (a) APPT electrodes and (b) Ag/AgCl gel electrodes at 0 h and after 9 h of wear.

**Figure S10.** SEM images of the surface morphology of the APPT sensor: (a) overall surface morphology; (b) higher magnification image of a representative AgNW-enriched region, revealing the interconnected AgNW network.

**Figure S11**. Stress-strain curve of the APPT sensor.

**Figure S12**. Variations of the resistance of APPT sensor with the strain.

**Figure S13**. Comparison between experimental and predicted values of conductivity, elongation, adhesion, and gauge factor for the APPT sensor.

**Figure S14.** Human-machine interface application set up.

**Reference**

[1] Noh, E.; Um, H.; Park, H. J.; Kim, M.; Kim, M.; Lee, S.; Lee, H. W.; Lee, J.; Lee, B. H. *Advanced Healthcare Materials* **2025**, *14* (24).

[2] Liu, Y.; Cheng, Y.; Shi, L.; Wang, R.; Sun, J. *ACS Applied Materials & Interfaces* **2022**, *14* (10), 12812–12823.

[3] Chen, M.; Chen, T.; Zhang, Y.; Fang, W.; Wenxuan Evelyn Li; Li, T.; Popovic, M. R.; Naguib, H. E. *Advanced functional materials* **2024**, *34*, 2403721.

[4] Zhou, X.; Rajeev, A.; Subramanian, A.; Li, Y.; Rossetti, N.; Natale, G.; Lodygensky, G. A.; Cicoira, F. *Acta Biomaterialia* **2022**, *139*, 296–306.

[5] Song, S.; Qi, L.; Lang, C.; Niu, H.; Zhou, H.; Niu, L.; Sun, Z.; Fang, J. *Applied Materials Today* **2026**, *48*, 102999.

[6] Niu, X.; Gao, X.; Wang, T.; Wang, W.; Liu, H. *ACS Applied Materials & Interfaces* **2022**, *14* (29), 33861–33870.

[7] Xing, L.; Wang, X.; Li, M.; Jia, Y.; Yang, G.; Liu, C.; Shen, C.; Liu, X. *Advanced Nanocomposites* **2024**, *1* (1), 171–179.

[8] Hong, S.; Park, T.; Lee, J.; Ji, Y.; Walsh, J.; Yu, T.; Park, J. Y.; Lim, J.; Benito Alston, C.; Solorio, L.; Lee, H.; Kim, Y. L.; Kim, D. R.; Lee, C. H. *ACS Sensors* **2024**, *9* (2), 662–673.

[9] Sun, Y.; Cheng, Y.; Shi, L.; Sun, J.; Chen, S.; Wang, R. *Advanced Functional Materials* **2024**, *34* (34).

[10] Li, R.; Zhang, H.; Li, L.; Zhang, B.; Du, X.; Shao, W.; Qian, X.; Cao, Y.; Liu, Z. *Journal of Materials Chemistry A* **2025**, *13* (18), 12988–12997.

[11] Shi, W.; Li, H.; Chen, J.; Ching, Y. C.; Chuah, C. H.; Xu, C.; Liu, M.; Zhang, J.; Ching, K. Y.; Liang, Y.; Li, G.; Tang, W. *Advanced Science* **2024**. *11*, 2404451

[12] Fatemeh Shahmoradi Ghaheh, Milad Razbin, M. Tehrani, L. Zolfipour, Mehdi Sadrjahani, *Scientific Reports* **2024**, *14*.

[13] B. Zhang, J. Lin, L. Du, L. Zhang, *Polymers* **2023**, *15*, 2224.

[14] M. A. Nielsen, *Neural Networks and Deep Learning.*, Determination Press, **2015**.

[15] L. V. Fausett, *Fundamentals of Neural Networks : Architectures, Algorithms, and Applications*, Pearson Education, New Delhi, **2008**.

[16] A. Rabienataj, S. M. Mousavi, Milad Razbin, M. Li, *Thermal Science and Engineering Progress* **2024**, *54*, 102795.

[17] D. Goldberg, *Genetic Algorithms : The Design of Innovation*, Springer, New York ; Berlin, **2007**.

[18] F. Li, X. Chen, P. Xu, Z. Fan, Q. Wang, C. Lyu, Q. Zhang, H. Yu, H. Wu, *Thin-walled structures* **2023**, *187*, 110752.
